# Metabolomic Profiles for Primary Progressive Multiple Sclerosis Stratification and Disease Course Monitoring

**DOI:** 10.3389/fnhum.2018.00226

**Published:** 2018-06-04

**Authors:** Daniel Stoessel, Jan-Patrick Stellmann, Anne Willing, Birte Behrens, Sina C. Rosenkranz, Sibylle C. Hodecker, Klarissa H. Stürner, Stefanie Reinhardt, Sabine Fleischer, Christian Deuschle, Walter Maetzler, Daniela Berg, Christoph Heesen, Dirk Walther, Nicolas Schauer, Manuel A. Friese, Ole Pless

**Affiliations:** ^1^Metabolomic Discoveries GmbH, Potsdam, Germany; ^2^Institut für Biochemie und Biologie, Universität Potsdam, Potsdam, Germany; ^3^Bioinformatik, Max-Planck-Institut für Molekulare Pflanzenphysiologie, Potsdam, Germany; ^4^Zentrum für Molekulare Neurobiologie Hamburg, Institut für Neuroimmunologie und Multiple Sklerose, Universitätsklinikum Hamburg-Eppendorf, Hamburg, Germany; ^5^Klinik und Poliklinik für Neurologie, Universitätsklinikum Hamburg-Eppendorf, Hamburg, Germany; ^6^Neurodegenerative Erkrankungen, Hertie-Institut für klinische Hirnforschung, Eberhardt-Karls-Universität Tübingen, Tübingen, Germany; ^7^Department of Neurology, Christian-Albrechts-Universität zu Kiel, Kiel, Germany; ^8^Fraunhofer IME ScreeningPort, Hamburg, Germany

**Keywords:** untargeted metabolomics, biomarker, PPMS, MS neurodegeneration, LysoPC(20:0)

## Abstract

Primary progressive multiple sclerosis (PPMS) shows a highly variable disease progression with poor prognosis and a characteristic accumulation of disabilities in patients. These hallmarks of PPMS make it difficult to diagnose and currently impossible to efficiently treat. This study aimed to identify plasma metabolite profiles that allow diagnosis of PPMS and its differentiation from the relapsing-remitting subtype (RRMS), primary neurodegenerative disease (Parkinson’s disease, PD), and healthy controls (HCs) and that significantly change during the disease course and could serve as surrogate markers of multiple sclerosis (MS)-associated neurodegeneration over time. We applied untargeted high-resolution metabolomics to plasma samples to identify PPMS-specific signatures, validated our findings in independent sex- and age-matched PPMS and HC cohorts and built discriminatory models by partial least square discriminant analysis (PLS-DA). This signature was compared to sex- and age-matched RRMS patients, to patients with PD and HC. Finally, we investigated these metabolites in a longitudinal cohort of PPMS patients over a 24-month period. PLS-DA yielded predictive models for classification along with a set of 20 PPMS-specific informative metabolite markers. These metabolites suggest disease-specific alterations in glycerophospholipid and linoleic acid pathways. Notably, the glycerophospholipid LysoPC(20:0) significantly decreased during the observation period. These findings show potential for diagnosis and disease course monitoring, and might serve as biomarkers to assess treatment efficacy in future clinical trials for neuroprotective MS therapies.

## Introduction

Primary progressive multiple sclerosis (PPMS) affects a subgroup of multiple sclerosis (MS) patients and shows a highly variable disease progression with poor prognosis (Katz Sand, 2015). While patients rarely present with clinical relapses as present in the more common relapsing-remitting subtype (RRMS), progressive accumulation of disabilities is characteristic. These hallmarks of PPMS make it difficult to diagnose and treat (Ontaneda et al., 2017).

Diagnosis of MS is currently based on the revised McDonald criteria (Polman et al., 2011) that include clinical assessments and MRI. However, diagnosis, subtype stratification, or assessment of disease progression by imaging, clinical, and biological markers is not yet reliable (Tremlett et al., 2005; Koch et al., 2009; Stellmann et al., 2014). Due to their accessibility and overall stability, surrogate blood metabolite markers of neuronal injury could substantially improve our mechanistic understanding and our ability to quantify neurodegeneration in MS (Botas et al., 2015) and could be applied to neuroprotective treatment trials to assess efficacy (Fox et al., 2012; Friese et al., 2014). Untargeted metabolomics is a high-throughput technology that allows for simultaneous semi-quantitative measurements of various metabolite species in complex samples such as biological fluids (Patti et al., 2012). Therefore, this is a suitable technology to obtain a comprehensive view of the functional state of the human organism (Xiao et al., 2012; Want et al., 2013; Contrepois et al., 2015). In addition, the high sensitivity and reliability of this technology makes it suitable for monitoring metabolite changes over time (Floegel et al., 2011).

By analyzing blood plasma with high-resolution mass spectrometry (HRMS), our study set out to discover metabolic profiles specific for PPMS. We identified a panel of 20 metabolites which discriminated PPMS from RRMS, Parkinson’s disease (PD), or healthy control (HC) and one metabolite which consistently decreased during a 24-month PPMS disease course.

## Materials and Methods

### Standard Protocol Approvals, Registrations, and Patient Consents

All participants (healthy donors, patients, or guardians of patients) provided written informed consent and the study was approved by the local ethics committee (Board of Physicians, Hamburg, Nos. PV4405 and PV3961). The PD study was approved by the Ethics Committee of the Faculty of Medicine at the University of Tübingen (26/2007BO1 and 497/2009BO1).

### Patient Recruitment and Diagnosis

Primary progressive multiple sclerosis (*n* = 33) and RRMS (*n* = 10) patients and HC (*n* = 33) were recruited at the University Medical Center Hamburg-Eppendorf. The participants underwent clinical assessment and provided plasma samples independent of meals. All HC in the study consist of both spouses and volunteers, which were recruited based on providing ‘matched pairs’ to patients with regard to age and sex. All HC underwent clinical assessment and reported no known autoimmune or neurological disease. All MS patients fulfilled the revised 2010 McDonald criteria (Polman et al., 2011) and had an Expanded Disability Status Scale (EDSS) below 7.0. RRMS patients, PPMS patients, and HC were recruited as a cross-sectional cohort, while additional PPMS patients were participating in a prospective observational longitudinal cohort study with annual visits and sampling of biomaterial. PPMS and RRMS patients have been off medication for at least three months. PD (*n* = 40) patients and HC (*n* = 20) were recruited at the Faculty of Medicine of the University of Tübingen, following equal procedures for blood withdrawal, processing, and storage as reported for the MS patients. PD diagnosis was based on the UK Brain Bank Society’s criteria for PD (Hughes et al., 1992). Of note, 15 out of 40 PD patients received levodopa (L-Dopa) treatment. Non-MS/PD-specific medication was absent, was not reported or was specific to single patients included in the study (one RRMS patient reported L-thyroxine supplementation). No patient or healthy donor reported diabetes as a comorbidity. The demographic and clinical features of patients with MS, PD, and healthy controls (HCs) are summarized in **Table 1**. A detailed list of all participants investigated can be found in **Supplementary Table S1**. Inter-cohort age and gender (sex) dependencies have been determined by chi-square test (sex) and one-way ANOVA (age, disease duration (DD), and EDSS; **Supplementary Table S2**). Of note, all biosamples were collected between 8 and 12 am and donors were not fasted.

**Table 1:** Cohort statistics.

	**Cross-sectional cohorts**							
	**PPMS cohort A**		**PPMS cohort B**		**RRMS cohort**		**Parkinson’s cohort**	
	**PPMS**	**HC**	**PPMS**	**HC**	**RRMS**	**HC**	**PD**	**HC**

Number (*n*)	13	13	20	20	10	10	40	20
Females (*n*, %)	8 (61.5)	7 (70)	7 (35)	7 (35)	7 (70)	7 (70)	16 (60)	13 (65)
Age (year ±*sd*)	52.5 ± 8.7	51.8 ± 8.7	51 ± 7.3	51.3 ± 6.7	44.2 ± 9.9	48.0 ± 10.0	64.8 ± 8.94	65.9 ± 7.13
Disease duration (year ±*sd*)	8.7 ± 5.9		7 ± 7.3		3.2 ± 7.5		7.0 ± 2.92	
EDSS (mean ± *sd*), median [range]	4.3 ± 1.3, 3.75 [2.5–6.5]		3.7 ± 1.3, 3.5 [2.0–6.0]		2.4 ± 0.9, 2.25 [1.5–4.0]		–	–
T25FW (s, mean ± *sd*), median [range]	6.5 ± 1.8, 5.7 [4.5–9.4]		6.3 ± 3, 5.1 [3.9–15.5]		4.9 ± 1.8, 4.15 [3.3–8.8]		–	–
6MWT (m, mean ± *sd*), median [range]	356.6 ± 108.8, 375 [230–530]		407.3 ± 107, 407 [180–590]		n.a.		–	–
SDMT (mean ± *sd*), median [range]	-0.4 ± 1.4, 0 [-3.0–1.5]		-0.8 ± 1, -0.5 [-3.0–0.5]		-0.4 ± 1.3, 0 [-2.5–1.5]		–	–
Hoehn and Yahr stage (mean ± *sd*, [range])	–		–		–		1.3 ± 0.69, [1–3]	
UPDRS total score (mean ± *sd*, [range])	–		–		–		25.2 ± 11.98, [6–57]	

	**Longitudinal cohort (PPMS)**							
	**Month 0**		**Month 12**		**Month 24**			

Number (*n*)	15							
Females (*n*, %)	5 (33.3)							
Age (year ±*sd*)	54.9 ± 6.9							
Disease duration (year ±*sd*)	7.8 ± 5.4							
EDSS (mean ± *sd*), median [range]	3.8 ± 1.4, 3.5 [2.0–7.0]		3.9 ± 1.5, 3.5 [1.5–7.5]		3.9 ± 1.5, 3.5 [1.5–7.0]							
T25FW (s, mean ± *sd*), median [range]	6.3 ± 2.9, 5.6 [4.0–15.5]		6.5 ± 3.8, 5.1 [3.9–18.9]		6.3 ± 2.6, 5.6 [4.3–14.8]							
6MWT (m, mean ± *sd*), median [range]	391.9 ± 91.9, 423.5 [180–500]		398.6 ± 106.8, 430 [120–520]		375.3 ± 139.3, 420 [160–500]							
SDMT (mean ± *sd*), median [range]	-0.5 ± 1.1, -0.5 [-3.0–1.0]		-0.4 ± 1.0, -0.5 [-2.5–1.5]		-0.3 ± 1.0, -0.5 [-1.5–1.5]							

*Demographic and clinical features of PPMS patients, RRMS patients, PD patients and HCs in cross-sectional cohorts, PPMS patients and HCs in the longitudinal PPMS cohort. Abbreviations: MS, multiple sclerosis; HCs, healthy controls; EDSS, Expanded Disability Status Scale; T25FW, Timed-25-Foot Walk Test; 6MWT, Six Minute Walk Test; SDMT, Symbol Digit Modalities Test; PD, Parkinson’s disease; UPDRS, Unified Parkinson’s disease rating scale*.

### Human Sample Preparation

Peripheral blood was collected in S-Monovette^®^ 9 ml, K3 EDTA, 92 × 16 mm test tubes (Sarstedt, 02.1066.001) and centrifuged for 8 min at 1200 ×*g* at 4°C. The supernatant was centrifuged at 4°C for an additional 10 min at 4300 ×*g*. The supernatant of the second centrifugation step was aliquoted, shock frozen in liquid nitrogen and stored at -80°C until further analysis.

### LC/MS and MS/MS Analysis

The analysis workflow is summarized in **Supplementary Figure S1**. Plasma metabolites were extracted using 90% MeOH and 10% water spiked with internal standards with constant shaking for 15 min at 37°C (1000 rpm). Modified hydrophilic interaction chromatography (HILIC) was employed in combination with HRMS. Samples were pseudonymized twice and third-party concealment of the origin of respective specimens (HC or MS) was achieved by using uniquely coded vials. Samples were randomized on an Agilent 1290 UHPLC system (Agilent, Santa Clara, United States) with a ZIC-HILIC column (10 cm × 2.1 mm, 3 μm, Sequant, Merck) coupled to a high-resolution 6540 QTOF/MS detector (Agilent, Santa Clara, United States) operated in both positive and negative ESI mode in a detection range of 50–1700 *m*/*z* at 2 GHz in an extended dynamic range. The LC solvent consisted of (A) 95% 10 mM ammonium acetate with 5% acetonitrile pH 6 and (B) 95% acetonitrile with 5% 10 mM ammonium acetate with a multi-step gradient (15 min runtime: 5% B from start to 1 min, to 35% B at 8.5 min, to 95% B at 9.5 min, to 5% B at 12.01 min until 15 min). One microliter of sample was injected at 30°C column temperature and the flow rate was kept constant at 300 μl/min. Chromatographic peaks, signal reproducibility, and analyte stability were monitored by assessment of biological quality controls, which were analyzed periodically throughout the batches. The DualAJS ESI source was set to the following parameters: Gas temperature 200°C, drying gas 8 l/min, nebulizer 35 psi, sheath gas temperature: 350°C, sheath gas flow 11 l/min, VCAp 3.5 kV, and nozzle voltage of 0 V. Online calibration of the instrument was performed with the Agilent ESI-TOF Reference Mass Solution Kit. MS/MS spectra were acquired in positive and negative ionization modes in a data-dependent and targeted manner with fragmentation energies of 0, 10, 20, and 40 V, respectively. Precursor isolation windows varied between narrow (1.3 *m*/*z*), medium (4 *m*/*z*), and wide (9 *m*/*z*; performed with Agilent MassHunter software).

### Metabolomics Data Analysis

Raw data were converted to mzXML and chromatogram peaks extracted with XCMS (Smith et al., 2006), which were optimized by using the IPO R-package (Libiseller et al., 2015). Mzmatch.R was used for peak filtering based on minimum detectable intensity (2000), peak shape filtering (codadw > 0.9) and for the annotation of related peaks (Scheltema et al., 2011). Additional filtering was performed by excluding peaks with lower median peak intensities per group in biological samples compared to blanks (extraction solvent only). The remaining data were normalized based on multiple internal standards applying Normalization using Optimal selection of Multiple Internal Standards (NOMIS; Sysi-Aho et al., 2007) and Cross-Contribution compensating Multiple standard Normalization (CCMN; Redestig et al., 2009) normalization. IDEOM software was used^1^ (Creek et al., 2012) to eliminate noise and artifacts and for putative peak annotation by exact mass within ±10 ppm against the Metabolomic Discoveries (MD) in-house metabolite library in negative and positive ESI modes, respectively. The MD metabolite database consists of ∼100 k metabolites from various classes such as lipids, peptides, and amino acids. Metabolite identification was performed as previously reported (Stoessel et al., 2018). Briefly, to aid metabolite annotation, retention time prediction was applied (Creek et al., 2011). Identities were confirmed by available authentic standards (validation level 1) and MS/MS spectra matched with online databases (e.g., Metlin and MassBank; validation level 2) or with *in silico* fragmentation spectra (validation level 3) from Metfrag (Ruttkies et al., 2016), CFM-ID (Allen et al., 2014), and/or CSI:FingerID (Duhrkop et al., 2015). Precursor mass accuracy was set to 20 ppm and fragment accuracy to 0.01 Da. Quantification of each metabolite was calculated using the raw peak height. Respective MSMS fragments were used for peak identification (**Supplementary Table S3**). Extracted ion chromatograms (EICs) of the determined PPMS markers can be found in **Supplementary Figure S2**. To allow for inter cohort comparisons, different run days have been normalized to zero mean differences between biological quality controls for peak intensities of the PPMS marker panel. These quality controls were injected before every batch.

### Statistical Analysis

Univariate statistical analyses utilized a Welch’s *t*-test (*p* = 0.05) and multivariate analyses utilized the metabolomics R package (De Livera and Bowne, 2014). Univariate area under the curve (AUC) measures and 95% confidence intervals (CIs) using 500 bootstrappings were calculated utilizing MetaboAnalyst (Xia et al., 2015) and Rmisc R package (Hope, 2013). Class discrimination and membership prediction was performed for subgroups of PPMS cohorts A and B by using 70% of all individuals for model training and the remaining 30% for blinded model testing. To ensure reproducibility of these results, patients were randomly selected for model testing and training 100 times. Additional class discrimination and membership prediction was performed by training a four-component PLS-DA model with all individuals from the PPMS cohort A and validating it in the blinded PPMS cohort B. Performances were gauged by the measures AUC, positive predictive values (PPVs), negative predictive values (NPVs), *R*^2^*X*/*R*^2^*Y* (fraction of the variation of the *X*/*Y* variables explained by the mode), *Q*^2^(cum), and accuracy of the calculated model. The datasets were scaled (zero mean, unit variance) prior to model building. For model prediction and AUC analysis, R-packages ROCR (Sing et al., 2005) and caret (Kuhn et al., 2016) were used. Furthermore, ‘variable importance in the projection’ (VIP) scores were computed and Welch’s *t*-test was applied to determine discriminatory variables in the dataset. The VIP is an estimation of the importance of each variable in the projection used in the partial least square discriminant analysis (PLS-DA) model as a quantitative estimation of the discriminatory power of each individual feature. Variables with a VIP score of ≥1 were considered important in the PLS-DA model. To investigate for potential confounding factors, these putative markers were tested for sex dependencies (Welch’s *t*-test) in PPMS cohort A and PPMS cohort B, and for age dependencies in PPMS cohort A, PPMS cohort B, RRMS cohort, and the PD cohort by linear regression. Moreover, to test whether these metabolites were able to discriminate PPMS from RRMS and PD, all PPMS patients and PD patients were combined. For the comparison of PPMS vs. RRMS, all individuals of the PPMS cohort A were used in combination with all RRMS patients to balance for class differences and potential gender dependencies. PLS-DA models were built using a random split of 70% of all individuals for model training and the remaining 30% for blinded model testing for each comparison. Again, to ensure reproducibility of these results, this random split was performed 100 times. To ensure that the obtained AUC, PPV, and NPV and accuracy values were higher than by chance, subject labels were randomly shuffled 100 times in the comparisons PPMS cohort A vs. HC, PPMS cohort B vs. HC, PPMS cohort A combined with PPMS cohort B vs. PD and PPMS cohort A vs. RRMS and empirical *p*-values were calculated. Univariate *p*-values for the comparison of PPMS vs. PD vs. RRMS were obtained using one-way ANOVA followed by Tukey’s *post hoc* test, which includes *p*-value adjustment for multiple comparisons. Additional *p*-value adjustments for multiple metabolites were carried out by using Benjamini and Hochberg (1995) false discovery rate (FDR) adjustment. Moreover, all *p*-values from paired and unpaired Welch’s *t*-tests were corrected for multiple testing (FDR).

Partial least square discriminant analysis computations were performed using the mixOmics (Le Cao et al., 2015) and ROPLS R-package (Le Cao et al., 2009; Thevenot et al., 2015). Suitable markers were selected, which enabled discrimination between HC and PPMS patients in the longitudinal cohort and time points 0 month (baseline), 12 months and 24 months utilizing paired *t*-test statistics (*p* < 0.01). These levels were also compared to levels of HC from our PPMS cohorts and RRMS and PD patients using one-way ANOVA followed by Tukey’s *post hoc* test. Significant levels in MetaboAnalyst pathway analysis were based on hypergeometric tests and the pathway impact values determined by relative-betweenness centrality (Xia et al., 2015).

## Results

### Plasma Metabolites Distinguish PPMS Patients From Healthy Controls

**FIGURE 1:**
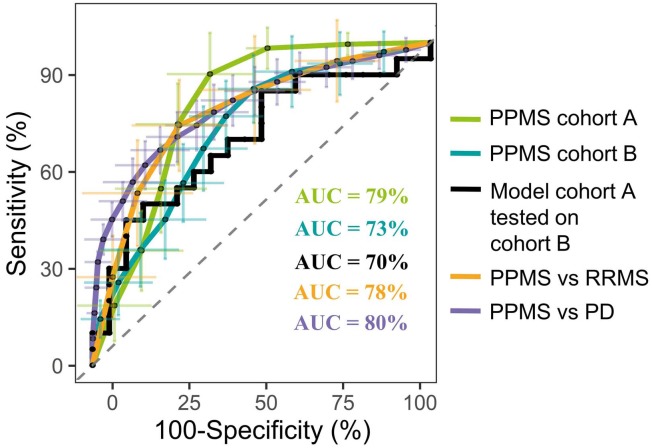
Representative PLS model shows good discrimination power in both cross-sectional PPMS cohorts. ROC curve of combined PLS model showing the true negative rate (specificity) vs. true positive rate (sensitivity). Green: Representative PLS model for cohort A (*n* (HC) = 13, *n* (PPMS) = 13), blue: representative PLS model for cohort B (*n* (HC) = 20, *n* (PPMS) = 20), black: PLS model of cohort B tested against blinded cohort A, orange: PLS model of PPMS patients from cohort A/B tested against RRMS patients (*n* = 10), purple: PLS model of PPMS patients from cohort A/B tested against PD patients (*n* = 40), and dashed gray line indicates 0 discrimination (AUC = 50%). Error bars indicate the standard deviation of the sensitivity and specificity over 100 iteration steps.

In order to identify metabolites that differentiate PPMS from HCs, we first employed a comparative untargeted metabolomics approach in plasma of HC (*n* *=* 33) and PPMS patients (*n* = 33), which we separated in two cohorts, cohort A (*n* = 13 per group, exploration cohort) and cohort B (*n* = 20 per group, validation cohort) (**Supplementary Figure S1A**). After individual measurements, we combined peak extraction procedures on the raw data for PPMS cohorts A and B to maximize the amount of overlapping mass peaks. Overall, 19,233 peaks in positive and 10,805 peaks in negative ionization modes were present in PPMS cohorts A and B. The within experiment technical and analytical variations were monitored based on periodic analysis of biological quality control samples in all cohorts. The median relative standard deviation (RSD) as an indicator for analytical reproducibility was <10%, which is within acceptable limits for metabolomics (Kirwan et al., 2014). Successive noise filtering, putative peak annotation, and combination of both ionization modes resulted in nomination of 534 putative metabolites (**Supplementary Table S4**). Identified metabolite classes and their prevalence are summarized in **Supplementary Figure S1B**. We analyzed the data by a supervised, multivariate classification technique (PLS-DA) to separately assess the overall segregation of the samples for PPMS cohorts A and B. Notably, this analysis allowed us to reproducibly separate HC from PPMS samples in both cross-sectional PPMS cohorts (**Supplementary Figures S1C,D**). In addition to this global and exploratory analysis that included all individuals, we tested whether the measured metabolites had a robust predictive value. Cohorts were split into training and test sets for deriving a PLS-DA-based model and to test its predictive performance, respectively (see section “Materials and Methods” for details). We used receiver operating characteristics (ROC) analysis of the training and testing model and determined a mean AUC of 79% (95% CI = 76–82), a mean PPV of 0.75 (95% CI = 0.72–0.78), and a mean NPV of 0.84 (95% CI = 0.80–0.88) for cohort A. In our PPMS cohort B, we could determine a mean AUC of 73% (95% CI = 72–74), a mean PPV of 0.69 (95% CI = 0.66–0.72), and a mean NPV of 0.70 (95% CI = 0.66–0.73). In addition, we used a PLS-DA model trained on PPMS cohort B data including all subjects to predict class memberships of the blinded PPMS cohort A. This cross-cohort test yielded an overall AUC of 70% (95% CI = 48–80), accuracy of 0.65, PPV of 0.65, and NPV of 0.65 (**Figure 1**). Random shuffling of subject labels in each cohort in this cross-cohort test indicates that the obtained model parameters were significantly larger than expected by chance (*p*-value ≤ 0.05). Detailed parameters for all tested models can be found in **Supplementary Table S5**.

### PPMS Patients Differentiate From Healthy Controls, RRMS, and PD Patients

**FIGURE 2:**
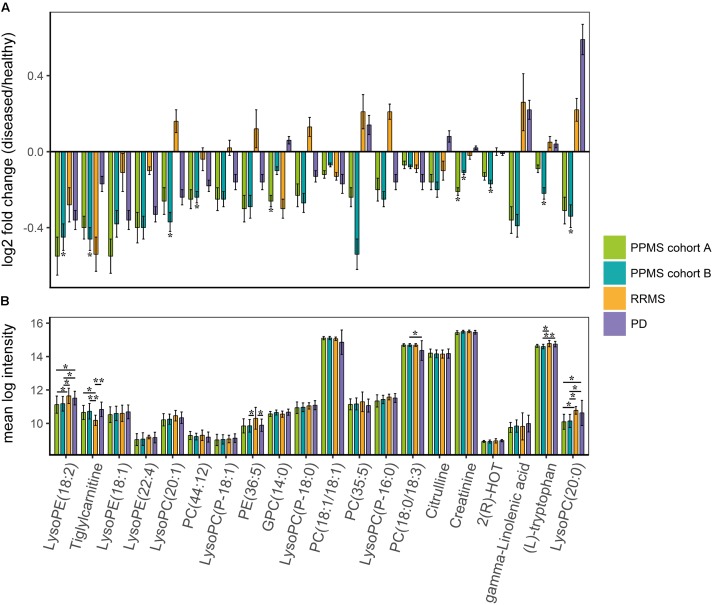
Metabolites contributing to the specific PPMS signature. Twenty features (i.e., metabolites) with VIP scores > 1 for components 1 and 2. **(A)** Summary with respective log2 fold changes (diseased/healthy). Green: PPMS cohort A, blue: PPMS cohort B, orange: RRMS, and purple: PD. **^∗^**Significant change (Welch’s *t*-test FDR ≤ 0.05), log2 fold change > 0: upregulation, log2 fold change < 0: downregulation. Error bars indicate standard deviations. **(B)** Comparison of PPMS patients from cohort A (green) and PPMS patients from cohort B (blue) to RRMS (orange) and PD (purple) patients. **^∗^**Significant change (one-way ANOVA with Tukey’s *post hoc* test *p* ≤ 0.05 (^∗∗^*p* ≤ 0.01), *p*-value adjusted for multiple comparisons).

Based on the PLS-DA models for each PPMS cohort, we extracted metabolites that contributed significantly to the differentiation between PPMS and HC in cohort A (*R*^2^*X* = 0.24, *R*^2^*Y* = 0.98, *Q*^2^(cum) = 0.68) and cohort B (*R*^2^*X* = 0.09, *R*^2^*Y* = 0.86, *Q*^2^(cum) = 0.45) by using VIP scores as a quantitative estimation of the discriminatory power of each individual metabolite. VIP scores were extracted for components 1 and 2 since they separated HC and PPMS almost to the same extent (**Supplementary Figures S1C,D**). Overall, 20 metabolites with a VIP score greater than 1 were reproducibly determined in both the PPMS cohorts A and B (11% of all metabolites with VIP ≥ 1 in PPMS cohort A and 12% of all metabolites with VIP ≥ 1 in PPMS cohort B) with univariate AUC values ≥60% (**Table 2** and **Supplementary Table S6**). Random shuffling of subject labels in each cohort yielded an empirical value of *p* = 0.004 for the number of 20 commonly found informative metabolites indicating that the overlap between cohorts is significantly larger than expected by chance. Of note, all of these metabolites were diminished in PPMS patients compared to HC and several were also significantly altered in either one or both PPMS cohorts (Welch’s *t*-test FDR ≤ 0.05; **Figure 2A**). To determine the specificity of these 20 metabolites for PPMS, we measured these metabolites in an independent cohort of 10 RRMS patients and 10 HC and an independent cohort of 40 PD patients and 20 HC. In our inter-disease comparison, we found levels of LysoPE(18:2) and LysoPC(20:0) to be significantly lower in PPMS compared to RRMS and PD (one-way ANOVA *p* ≤ 0.05 with Tukey’s *post hoc* test). Moreover, tiglylcarnitine was found to be significantly higher in PPMS compared to RRMS (one-way ANOVA *p* ≤ 0.05 with Tukey’s *post hoc* test; **Figure 2B** and **Supplementary Table S7**). Of note, these differences appeared to be only significant (FDR ≤ 0.05), when *p*-value correction was performed for comparison of multiple groups. Compared to HC, we found some of the metabolites to be consistently downregulated in all diseases analyzed, indicating a general signature of neurodegeneration, but also found clear disease-specific differences, e.g., a downregulation of the metabolites gamma-Linolenic acid, (L)-tryptophan, and LysoPC(20:0) in PPMS and an upregulation of these analytes in RRMS and PD (**Figure 2B** and **Supplementary Tables S8**, **S9**).

**Table 2:** Detailed information on significantly changed metabolites between HC and PPMS patients (A and B cohorts).

Proposed metabolite	Proposed formula	*m/z*	Mass error (ppm)	*p*-value A	*p*-value B	Log2 FC A	Log2 FC B	VIP comp1 A	VIP comp1 B	VIP comp2 A	VIP comp2 B	KEGG ID	Class	Validation level
Citrulline	C_6_H_13_N_3_O_3_	175.09587	1.05	0.137 0.156	**0.034** 0.052	-0.15	-0.20	1.22	1.56	1.13	1.32	C00327	Carboxylic acids and derivatives	1
Creatinine	C_4_H_7_N_3_O	113.05889	-0.19	**0.001 0.02**	**0.011 0.04**	-0.21	-0.11	2.6	1.85	2.37	1.47	C00791	Azoline	1
(L)-tryptophan	C_11_H_12_N_2_O_2_	204.0902	1.58	0.148 0.156	**0.004 0.023**	-0.09	-0.22	1.18	2.08	1.08	1.64	C00078	Indoles and derivatives	1
LysoPE(18:1)	C_23_H_46_NO_7_P	479.30081	-0.79	**0.028** 0.083	**0.044** 0.055	-0.55	-0.38	1.74	1.49	1.65	1.21	/	Glycerophospho ethanolamine	3
LysoPE(18:2)	C_23_H_44_NO_7_P	477.28562	0.18	**0.029** 0.083	**0.011 0.04**	-0.55	-0.45	1.74	1.84	1.66	1.48	/	Glycerophospho ethanolamine	3
LysoPE(22:4)	C_27_H_48_NO_7_P	529.31598	-1.62	0.161 0.161	**0.031** 0.052	-0.40	-0.40	1.15	1.58	1.05	1.39	/	Glycerophospho ethanolamine	3
LysoPC(P-16:0)	C_24_H_50_NO_6_P	479.33765	0.15	0.133 0.156	**0.033** 0.052	-0.20	-0.25	1.2	1.49	1.19	1.34	C04230	Glycerophos phocholine	3
LysoPC(P-18:0)	C_26_H_54_NO_6_P	507.3682	-1.34	0.142 0.156	**0.044** 0.055	-0.23	-0.26	1.34	1.58	1.15	1.29	C04230	Glycerophos phocholine	3
LysoPC(P-18:1)	C_26_H_52_NO_6_P	505.35284	-0.77	0.099 0.156	**0.032** 0.052	-0.25	-0.25	1.27	1.82	1.26	1.26	C04230	Glycerophos phocholine	3
PC(44:12)	C_52_H_80_NO_8_P	877.56643	4.87	0.118 0.156	**0.012 0.04**	-0.25	-0.24	1.18	1.71	1.17	1.44	C00157	Glycerophos phocholine	3
LysoPC(20:1)	C_28_H_56_NO_7_P	549.3787	-1.34	0.147 0.156	**0.019 0.048**	-0.26	-0.37	1.43	1.75	1.14	1.38	C04230	Glycerophos phocholine	3
LysoPC(20:0)	C_28_H_58_NO_7_P	551.39427	-1.48	0.079 0.156	**0.017 0.048**	-0.31	-0.34	1.24	1.57	1.34	1.42	C04230	Glycerophos phocholine	3
PE(36:5)	C_41_H_72_NO_8_P	737.49806	-2.03	0.066 0.156	0.08 0.094	-0.30	-0.29	1.5	1.3	1.4	1.03	C00350	Glycerophospho ethanolamine	3
PC(35:5)	C_43_H_76_NO_8_P	765.53003	-1.08	0.082 0.156	0.106 0.111	-0.24	-0.54	1.41	1.21	1.3	1.08	C00157	Glycerophos phocholine	3
PC(18:1/18:1)	C_44_H_84_NO_8_P	785.59391	0.57	**0.026** 0.083	0.088 0.098	-0.12	-0.07	1.77	1.27	1.62	1.11	C00157	Glycerophos phocholine	3
PC(18:0/18:3)	C_44_H_82_NO_8_P	783.57697	-1.07	0.139 0.156	**0.027** 0.052	-0.07	-0.08	1.21	1.62	1.24	1.35	C00157	Glycerophos phocholine	3
Tiglylcarnitine	C_12_H_21_NO_4_	243.14724	0.73	**0.018** 0.083	**0.003 0.027**	-0.40	-0.46	1.87	2.15	1.81	1.77	/	Fatty Acyl	3
2(R)-HOT	C_18_H_30_O_3_	294.22165	7.31	**0.013** 0.083	**0.001 0.02**	-0.13	-0.17	1.98	2.32	1.8	1.94	C16342	Lineolic acids and derivatives	3
GPC(14:0)	C_46_H_78_NO_7_P	787.54959	-2.53	**0.003 0.03**	0.12 0.12	-0.26	-0.10	2.25	1.16	2.06	1.06	/	Glycerophos phocholine	3
Gamma- Linolenic acid	C_18_H_30_O_2_	278.22449	-0.32	0.127 0.156	**0.041** 0.055	-0.36	-0.39	1.25	1.51	1.16	1.19	C06426	Lineolic acids and derivatives	2

*Overlapping significant ions detected between HC and PPMS patients. Proposed metabolite: Proposed metabolite for each ion. Proposed formula: Formulas obtained using m/z data with IDEOM. m/z: mass/charge ratio corrected for the mass of one proton. Mass error (ppm): [(m/z(observed) -m/z(theoretical)] × 1E + 6. Log2 fold change (FC): relative abundance of mean of corresponding ion in PPMS patients compared to the mean of HC. oxp-value: Value for unpaired Welch’s oxt-test, first oxp-value is unadjusted, second oxp-value adjusted for multiple testing (FDR). Metabolites with oxp-value < 0.05 are highlighted in bold*.

Multivariate analysis of these 20 metabolites for the discrimination compared to RRMS (*R*^2^*X* = 0.79, *R*^2^*Y* = 0.93, *Q*^2^(cum) = 0.93) led to a mean AUC of 78% (95% CI = 75–82), mean PPV of 0.72 (95% CI = 0.68–0.76), mean NPV of 0.77 (95% CI = 0.73–0.81), and a mean accuracy of 0.68 (95% CI = 0.65–0.71). Multivariate discrimination relative to PD (*R*^2^*X* = 0.69, *R*^2^*Y* = 0.56, *Q*^2^(cum) = 0.56) to a mean AUC of 80% (95% CI = 78–82), mean PPV of 0.75 (95% CI = 0.73–0.77), mean NPV of 0.71 (95% CI = 0.68–0.73), and a mean accuracy of 0.71 (95% CI = 0.70–0.73; **Figure 1**). Again, random shuffling of subject labels in each comparison indicates that the obtained model parameters are significantly larger than expected by chance (*p* ≤ 0.05). Overall, the identified markers indicate a PPMS-specific plasma signature.

### PPMS-Specific Metabolites Profile Suggest Alterations in Glycerophospholipid Metabolism

**FIGURE 3:**
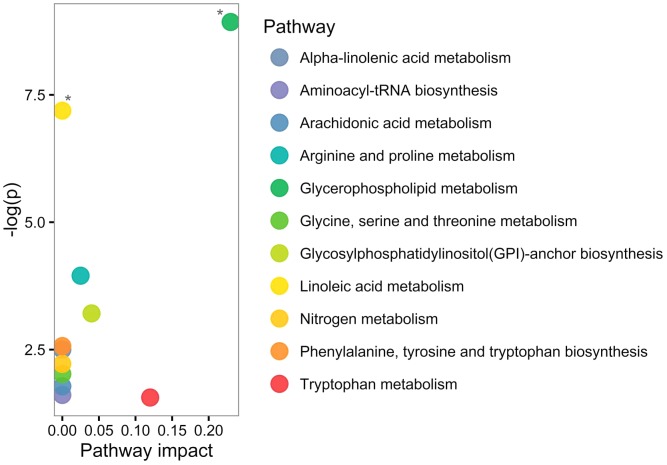
Pathway analysis of altered metabolites using MetaboAnalyst shows perturbations in glycerophospholipid and linoleic acid pathways. Twenty significantly deregulated metabolites identified were subjected to MetaboAnalyst (Xia et al., 2015) to assess association of each metabolite with defined pathways. *X*-axis presents pathway impact values (based on relative-betweenness centrality) and the *Y*-axis presents the respective *p*-values (based on hypergeometric test). ^∗^*p*-value ≤ 0.05 after FDR correction. The largest and significant impact was found in glycerophospholipid and linoleic acid pathways.

Based on human metabolome database (HMDB; Wishart et al., 2013) and KEGG identifiers of the 20 altered PPMS-specific metabolites, we set out to perform pathway analysis utilizing MetaboAnalyst (Xia et al., 2015). Overall, our untargeted metabolic profiling revealed several significant perturbations, which allowed identification of multiple significant biochemical pathways in particular in the glycerophospholipid pathway and linoleic acid metabolism (FDR ≤ 0.05; **Figure 3**).

### Levels of LysoPC(20:0) Decline During the Disease Course of PPMS

**FIGURE 4:**
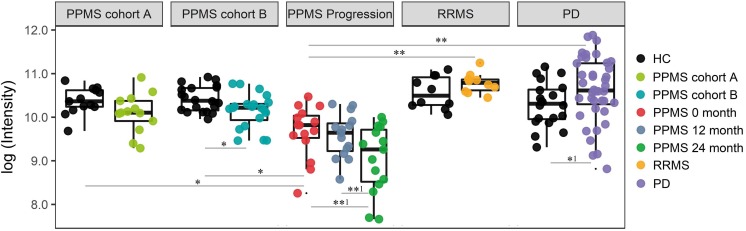
Intensity levels for glycerophospholipid LysoPC(20:0) show relative differences in abundance in patients during PPMS disease course. *PPMS cohort A*: Decreased LysoPC(20:0) levels in PPMS patients compared to HC, black: HC (*n* = 13), green: PPMS patients (*n* = 13). *PPMS cohort B*: LysoPC(20:0) levels lower in PPMS patients compared to HC, black: HC (*n* = 20), blue: PPMS patients (*n* = 20). *PPMS progression*: Data from the disease course cohort showing decreasing levels of LysoPC(20:0) (0, 12, and 24 months: *n* = 15), red: baseline (0 month), dark blue: PPMS 12 months, and green: PPMS 24 months. *RRMS*: Black: HC (*n* = 10), yellow: RRMS patients (*n* = 10). *PD*: black: HC (*n* = 20), purple: PD patients (*n* = 40). LysoPC(20:0) is significantly elevated in PD and elevated in RRMS patients compared to HC. ^∗^FDR ≤ 0.05, ^∗∗^FDR ≤ 0.01, ^∗∗1^FDR ≤ 0.1, ^∗1^*p*-value ≤ 0.05 without FDR correction.

Finally, we investigated the ability of the determined metabolite profile to monitor disease course by analyzing a longitudinal PPMS cohort (*n* = 15) over 24 months. Patients were clinically investigated and their plasma was sampled at baseline, after 12 and 24 months. Notably, 18 of 20 determined metabolites showed no significant change between baseline (0 months), 12 months, and 24 months disease course (paired *t*-test *p* < 0.01). However, the glycerophospholipid annotated as LysoPC(20:0) showed a significant reduction when comparing 12 months and 24 months values (*p*-value = 0.0034, FDR-adjusted *p*-value = 0.0576; paired *t*-test), fold change (12 months/24 months) = 1.59) or baseline (0 month) and 24 months values (*p*-value = 0.0044, FDR-adjusted *p*-value = 0.0888 (paired *t*-test), fold change (0 month/24 months) = 1.84; **Figure 4** and **Supplementary Table S10**). Therefore, our data show a strong association of LysoPC(20:0) with the PPMS disease course over time. Moreover, LysoPC(20:0) baseline values were found significantly lower compared to HC in PPMS cohort A (*p*-value = 0.0316), HC in PPMS cohort B (*p*-value = 0.0036), RRMS patients (*p*-value = 7.27 × 10^-5^), and PD patients (*p*-value = 1.49 × 10^-6^, one-way ANOVA and Tukey’s *post hoc* test). Since none of the recorded clinical measures (EDSS, SDMT, and walking tests) showed a significant change over the investigated time of the PPMS disease course (ANOVA *p*-value ≤ 0.05), we were restricted in performing further correlative analysis. Recent evidence, however, suggests that significant changes in clinical parameters are unlikely to manifest during a 24-month time period in PPMS (Signori et al., 2017). We could not find a correlation between LysoPC(20:0) levels and corresponding patient’s age (linear regression, *p*-value = 0.22).

### Analysis of Confounding Factors

A number of tests were performed to rule out confounding factors in our analysis. We reduced the sex bias in MS (Voskuhl and Gold, 2012) by comparing equal sex distributions in the different cohorts (HC, PPMS, RRMS, PD, and longitudinal cohort). In addition, we compared strictly age-matched cohorts. RRMS patients are on average younger than PPMS patients and the cohorts analyzed in this study represent ‘real life’ patients who visit our outpatient clinics. Nevertheless, the age was not significantly different between PPMS cohorts A and B and the RRMS cohort (**Supplementary Table S8**). Therefore, we decided not to normalize for age differences between these two cohorts. Furthermore, we tested for an age-related bias in the analysis, which we could refute for LysoPC(20:0) (age difference not significant according to ANOVA, *p* ≤ 0.05, **Supplementary Figure S3**). Furthermore, age-related dependencies could only be identified for gamma-Linolenic acid (negative correlation) and LysoPC(20:1) (positive correlation; **Supplementary Figure S3**). We also tested for any sex-related bias in this analysis. Overall, several metabolites appeared to show significantly (ANOVA, *p* ≤ 0.05 with Tukey’s *post hoc* test) different levels between males and females in PPMS cohorts A and B (**Supplementary Figure S4**). To compare equal gender distributions among groups for our PPMS vs. RRMS comparison, we used PPMS patients from PPMS cohort A when tested for specificity against RRMS patients. Since the individuals in our PD cohort were significantly older (ANOVA *p* ≤ 0.05) than our PPMS patients, we also tested for potential age-related trends of the identified 20 metabolites in the PD cohort. We only observed significant negative correlations (linear model *p* < 0.05) for gamma-Linolenic acid and (L)-tryptophan (**Supplementary Figure S5**). Since these metabolites do not show any significant differences between PPMS and PD, we decided not to normalize for these effects. In addition, we could not identify any significant age-related trends in the RRMS cohort (**Supplementary Figure S6**). Finally, even though 15 out of 40 PD patients received L-Dopa, this treatment showed no significant effect when compared to treatment-naive PD patients on any of our 20 PPMS metabolites (data not shown, Welch’s *t*-test *p*-value ≤ 0.05).

## Discussion

### PPMS Metabolite Panel Discriminates From HC, RRMS, and PD

Primary progressive multiple sclerosis is a clinically highly variable inflammatory neurodegenerative disease and is associated with a poor prognosis and continuous accumulation of neurological symptoms and disabilities (Antel et al., 2012). Currently, no reliable molecular biomarker-based diagnosis or monitoring of disease course is available. These would, however, be of utmost importance for the development of novel therapeutic options tackling PPMS-associated neurodegeneration.

Untargeted metabolomics allows for the simultaneous identification and quantification of a wide range of metabolites in biofluid samples which can be used to differentiate between ‘healthy’ and ‘diseased’ and can lead to the discovery of pathways involved in pathogenesis. The generated PLS-DA models showed a moderate discriminatory power to segregate PPMS patients and healthy individuals. The PLS-DA model trained with PPMS cohort B was tested on blinded cohort A and achieved an AUC of 70%. While significantly better than randomly expected, also corroborated by the significant correlation of metabolite VIP scores between the two cohorts (**Figure 1**), this performance does not yet allow to conclude general diagnostic utility of the PLS model. Aside from the limitations in set size, this may be indicative of unique metabolic signatures in each cross-sectional PPMS cohort most likely caused by cohort effects such as differences in sample storage duration and physiological variance between individuals. Also, performing sampling at defined time points of the day, preferably from fasted individuals, would have been optimal for this study but is difficult to achieve in clinical practice. Nonetheless, a subset of 20 significantly altered metabolites was determined reproducibly with a good separation between the investigated PPMS patients and HC, RRMS, and PD. Thus, they can be considered PPMS specific.

A potential medication bias in the study presented was eliminated by (a) including treatment naïve patients (RRMS samples), by (b) including PPMS patients which have been off medication for at least three months prior to sampling, and by (c) excluding patients with known metabolic diseases in our clinical cohorts. Based on the given medication, it was not possible to build a significant PLS-DA model indicating that medication was no confounding factor in our identified marker profile. Moreover, analysis for our different PPMS, RRMS, and PD cohorts as well as corresponding controls was not biased as equal procedures for blood withdrawal, processing, storage, analytics, data acquisition, and analysis were applied to all samples. Overall, these results indicate that sex, gender, and medication appear to have no effects as confounding factors on our PPMS marker profile. Differences in age appear to be disease specific. Even though, our selection of 20 significantly changed metabolites overlapping between both PPMS cohorts appears to be lower than expected by chance, the set of the top 25 metabolites in both PPMS models (i.e., a larger candidate set than the 20 metabolites selected for stricter reasons and now based on their weighted VIP scores alone) reaches significance (*p*-value = 0.0182). Thus, applying a multivariate prediction model (PLS-DA), the metabolites detected relevant in one cohort proved informative in the second cohort as well.

### Pathway Alterations in PPMS

Even though the main objective of our study was to identify novel metabolite-based biomarkers for PPMS diagnosis that change during disease course our study also informs on the pathophysiology of the disease by the identification of alterations in distinct metabolite pathways (**Supplementary Figure S7**). We identified reduced levels of five phosphatidylcholines (PC), four lysophosphatidylcholines (lysoPC), and one glycerophosphatidylcholine (GPC) in PPMS patients. PC lipids and a large range of phospholipids are represented in the majority of eukaryotic cellular membranes. The PLA_2_ superfamily of enzymes catalyzes the hydrolysis of *sn*-2 ester bonds of glycerophospholipids (including PCs), resulting in the production of free fatty acids and lysophospholipids (including lysoPCs; Murakami and Kudo, 2002). PLA_2_ products including lysoPCs are involved in a multitude of downstream pathways, orchestrating signal transduction via second messenger generation, driving biosynthesis of inflammatory mediators, neurotransmitter release, cell growth, differentiation, and apoptosis (Farooqui, 2009). Evidence of a deregulation of PLA_2_ and its products in disease has accumulated over the last decades and has been reported for several diseases including primary neurodegenerative diseases, such as Alzheimer’s disease (AD), PD, amyotrophic lateral sclerosis (ALS), ischemia, and spinal cord injury (Farooqui and Horrocks, 2006). Interestingly, our findings of reduced lysoPC and PC species are supported by previous reports of an altered phospholipid metabolism in serum of MS patients (Del Boccio et al., 2011). Since both concentration and activity of lipoprotein-associated phospholipase A2 (Lp-PLA_2_) are similar in plasma of PPMS patients and controls (Sternberg et al., 2012), an attractive hypothesis is a disease-specific functional impairment of brain-specific PLA_2_, which could account for lysoPC depletion. Alternatively, lysoPC species are generated by the enzyme lecithin:cholesterol acyltransferase (LCAT), which catalyzes the transfer of a fatty acyl residue from the *sn*-2 position of phosphatidylcholine to the 3-beta-hydroxy group of cholesterol, resulting in the formation of cholesteryl esters. Early studies, however, have suggested an increased LCAT activity in plasma of progressive MS patients compared to HC which is contradictory to our findings (Andreoli et al., 1973).

Our untargeted metabolomics approach revealed some additional statistically significant changes in 2(R)-HOT and gamma-Linolenic acid, products of linoleic acid metabolism. These metabolites are essential for the production of several omega-3 fatty acids, which in turn inhibit the actions of arachidonic acid and its pro-inflammatory derivatives. A randomized, double-blind, placebo-controlled, proof-of-concept clinical trial treating RRMS patients with omega-3 fatty acids supplementation has reached the primary endpoint (reduction in annual relapse rate; Pantzaris et al., 2013). These findings are in line with the hypothesis of a deregulated linoleic acid metabolism in MS patients.

Of note, a deregulation of (L)-tryptophan in cerebrospinal fluid (CSF) and plasma of MS patients was reported several times and is consistent with our findings (Monaco et al., 1979; Rudzite et al., 1996; Sandyk, 1996; Cocco et al., 2016). (L)-tryptophan is a substrate in the kynurenine pathway which is of particular interest in neuroinflammatory diseases because it contributes to immune regulation and generates both neurotoxic and neuroprotective mediators. Evidence of the link between the kynurenine pathway and MS pathogenesis is already well established and targeting crucial enzymes in this pathway could be a future treatment option (Lovelace et al., 2016; Lim et al., 2017).

In addition, we found depleted levels of citrulline. Citrulline plays a role in arginine biosynthesis and is also generated by arginine conversion. Perturbations in protein citrullination, in particular in citrullination of myelin basic protein (MBP), have been reported in MS and enzymes responsible for arginine citrullination (peptidyl arginine deiminases, PADs) have been discussed as potential molecular targets for MS therapy (Yang et al., 2016). To what extend freely circulating citrulline in the peripheral blood mirrors aberrantly citrullinated proteins in the central nervous system remains speculative at this point.

### LysoPC(20:0) Declines Over PPMS Disease Course

Notably, almost all metabolites that led to a discrimination between HC and PPMS patients showed decreased concentrations in the latter and were stable over the investigated time course (24 months). The lipid annotated as LysoPC(20:0) is a notable exception since it significantly declines over the 24-month disease course. In contrast, elevated levels of LysoPC(20:0) were found in RRMS, PD patients, and compared to their matched HCs indicating PPMS specificity. Taken together, the significantly altered metabolite species reported here could allow for their future diagnostic application soon after disease onset. Whether LysoPC(20:0) could serve as a surrogate marker for DD and/or for the extent of neurodegeneration remains to be explored in a prospective cohort, preferably also including a longitudinal HC study arm. Moreover, to further investigate LysoPC(20:0) as a potential progression marker, its levels should be fully quantified in PPMS patients and HC over time.

## Conclusion

In conclusion, metabolic profiling of plasma was found to be a promising technology for non-invasive diagnosis of PPMS. The results of this study led to the identification of a potential marker panel for PPMS diagnosis, which is decreased in plasma concentration compared to HC, RRMS, and PD. This also provides insights into specifically altered glycerophospholipid and linoleic acid metabolisms in PPMS patients. In future studies, it will be necessary to investigate the discriminatory power of the reported potential PPMS marker(s) in specimen of equally well-characterized larger clinical cohorts. Our results open a new line of investigation to specifically monitor phosphatidylcholine species as potential biomarkers for disease diagnosis and disease course progression.

## Data Availability Statement

### Restrictions Apply to the Datasets

The patient-associated datasets of this study cannot be made publicly available because of the nature of the study (as stated in the ethical documentation of the Institute for Neuroimmunology und Multiple Sclerosis and the Neuro Biobank Tübingen). Requests to access the datasets should be directed to the corresponding author.

## Author’s Note

Part of this work has been submitted for patent registration at the European Patent Organisation (EPO), No. 17159341.1.

## Author Contributions

DS and J-PS have analyzed and interpreted data, drafted/revised the manuscript for content, and have contributed to study concept and design. AW, SCR, KS, and DW have analyzed and interpreted data and have revised the manuscript for content. BB and SH have analyzed and interpreted data. SR and CD have analyzed data. SF, WM, DB, and CH have contributed to study concept and design. NS revised the manuscript for content and has contributed to study concept and design. MF and OP have interpreted data, drafted/revised the manuscript for content, and have contributed to study concept and design.

## Conflict of Interest Statement

The authors declare that the research was conducted in the absence of any commercial or financial relationships that could be construed as a potential conflict of interest.

## Footnotes

1 http://mzmatch.sourceforge.net/ideom.php

## Supplementary Material

FIGURE S1 Workflow and summary of metabolomic profiling. **(A)** General workflow in data analysis. **(B)** Pie chart illustrating the percentage of putatively identified metabolites from each metabolite class, classified according to KEGG, Lipidmaps and HMDB. In total 534 metabolites were analysed. **(C)** PLS-DA scores plot of all 534 metabolites in HC cohort A (*n* = 13) (black) and PPMS cohort A (*n* = 13) (green). **(D)** PLS-DA scores plot 534 metabolites identified in the HC cohort B (*n* = 20) (black) and PPMS cohort B (*n* = 20) (blue). Data in **(C,D)** show clear a separation between PPMS patients and HC. Ellipses assume a multivariate Gaussian distribution (2 sigma).

FIGURE S2 Extracted ion chromatograms of metabolites contributing to the specific PPMS signature.

FIGURE S3 Linear model fit of PPMS marker levels and age of all analyzed individuals in PPMS cohorts A and B. Utilized linear model to fit correlation between PPMS marker levels and ageing. Black: Linear model for all data points, light red: linear model for HC cohort A, green: linear model for HC cohort B, blue: linear model for PPMS cohort A, purple: linear model for PPMS cohort B. Grey areas around the lines indicate the 0.95 confidence interval. Metabolites with significant (*p*-value < 0.05) age and level correlations are highlighted with a red rectangle.

FIGURE S4 PPMS marker levels and sex dependency in all individuals analyzed. Utilized one way ANOVA with Tukey’s *post hoc* test to compute differences between PPMS marker levels and sex in all groups. ^∗^Metabolites with significantly different levels between males and females (*p*-value < 0.05), ^∗∗^Metabolites with significantly different levels between males and females (*p*-value < 0.01).

FIGURE S5 Linear model fit of PPMS marker levels and age of all individuals analyzed in the PD cohort. Utilized linear model to fit correlation between PPMS marker levels and ageing. Black: Linear model for all data points, green: linear model for HC, purple: linear model for PD. Grey areas around the lines indicate the 0.95 confidence interval. Metabolites with significant (*p*-value < 0.05) correlations between age and level are highlighted with a red rectangle.

FIGURE S6 Linear model fit of PPMS marker levels and age of all analyzed individuals in the RRMS cohort. Utilized linear model to fit correlation between PPMS marker levels and ageing. Black: Linear model for all data points, green: linear model for HC, orange: linear model for RRMS. Grey areas around the lines indicate the 0.95 confidence interval. Metabolites with significant (*p*-value < 0.05) age and level correlations are highlighted in a red rectangle.

FIGURE S7 Pathway analysis of identified changes in PPMS patients compared to healthy controls (HC). Pathway information retrieved from KEGG. Significantly altered metabolites are highlighted in dark red rectangles including the corresponding KEGG ID. Grey font indicates involved enzymes, dashed grey line indicates additional reactions which are not displayed. The substrates and products of reactions are shown in grey rectangular boxes. Green boxes indicate possible cellular function of observed changes in each pathway, decreased levels of metabolites compared to HC are indicated by blue arrows. Possible products and substrates are only partially displayed. Lysophosphatidylcholines lysoPE(18:1), lysoPE(18:2), and lysoPE(22:4) without reported KEGG ID (proposed KEGG ID C00438).

TABLE S1 Cohorts. Patient information from investigated RRMS, PPMS, PD cohorts including age and gender. A disease duration of 0 corresponds to less than 1 year (^∗^.xlsx file).

TABLE S2 Inter-cohort dependencies determined by chi-square test for gender and one-way ANOVA for age, EDSS and disease duration (DD). Comparisons with significant *p*-values (*p*-value < 0.05) are highlighted in bold red.

TABLE S3 MS/MS fragments used for PPMS marker identification. Identities confirmed by available authentic standards (validation level 1). MS/MS spectra were matched against online databases such as Metlin and MassBank (validation level 2) or against *in silico* fragmentation spectra (validation level 3) retrieved from Metfrag, CFM-ID and/or CSI:FingerID with precursor mass accuracy of 20 ppm and fragment accuracy of 0.01 Da.

TABLE S4 Plasma metabolites identified in PPMS cohorts A and B (^∗^.xlsx file).

TABLE S5 Model parameters for the tested models and their corresponding p-values after sample-label permutation test.

TABLE S6 Metabolites with significant changes between HC and PPMS patients (A and B cohorts) their corresponding AUC values and VIP score ranks for component 1 and 2. Univariate AUCs and 95% CI (500 bootstrapping) calculated using MetaboAnalyst.

TABLE S7 PPMS marker in PPMS patients compared to RRMS and PD patients. First p-value is determined by one-way ANOVA with Tukey’s *post hoc* with correction for multiple testing per metabolite. Metabolites with *p*-values < 0.05 are highlighted in bold green. Second *p*-value has been corrected (FDR) for all comparison including all metabolites.

TABLE S8 PPMS marker measured in RRMS, intensity levels in the RRMS cohort. *P*-values determined by Welch’s *t*-test, *p*-value adjustment for multiple comparison using Benjamini and Hochberg (FDR) adjustment (^∗^.xlsx file).

TABLE S9 PPMS marker measured in PD, intensity levels in the PD cohort. P-values determined by Welch’s *t*-test, *p*-value adjustment for multiple comparison using Benjamini and Hochberg (FDR) adjustment (^∗^.xlsx file).

TABLE S10 Metabolite levels with significant changes between HC and PPMS patients measured in the longitudinal PPMS cohort (^∗^.xlsx file).
