# Supplementary material for: Metabolomic Profiles for Primary Progressive Multiple Sclerosis Stratification and Disease Course Monitoring
Source: Front Hum Neurosci. 2018 Jun 4;12:226. doi: 10.3389/fnhum.2018.00226 (PMC5994544; doi:10.3389/fnhum.2018.00226)
Supplement: FIGURE S1 — Workflow and summary of metabolomic profiling. (A) General workflow in data analysis. (B) Pie chart illustrating the percentage of putatively identified metabolites from each metabolite class, classified according to KEGG, Lipidmaps and HMDB. In total 534 metabolites were analysed. (C) PLS-DA scores plot of all 534 metabolites in HC cohort A (n = 13) (black) and PPMS cohort A (n = 13) (green). (D) PLS-DA scores plot 534 metabolites identified in the HC cohort B (n = 20) (black) and PPMS cohort B (n = 20) (blue). Data in (C,D) show clear a separation between PPMS patients and HC. Ellipses assume a multivariate Gaussian distribution (2 sigma). [file Data_Sheet_1.docx]

**Metabolomic profiles for primary progressive multiple sclerosis stratification and disease course monitoring**

**Daniel Stoessel, Jan-Patrick Stellmann, Anne Willing, Birte Behrens, Sina C. Rosenkranz, Sibylle C. Hodecker, Klarissa H. Stürner, Stefanie Reinhardt, Sabine Fleischer, Christian Deuschle, Walter Maetzler, Daniela Berg, Christoph Heesen, Dirk Walther, Nicolas Schauer, Manuel A. Friese, Ole Pless**

**
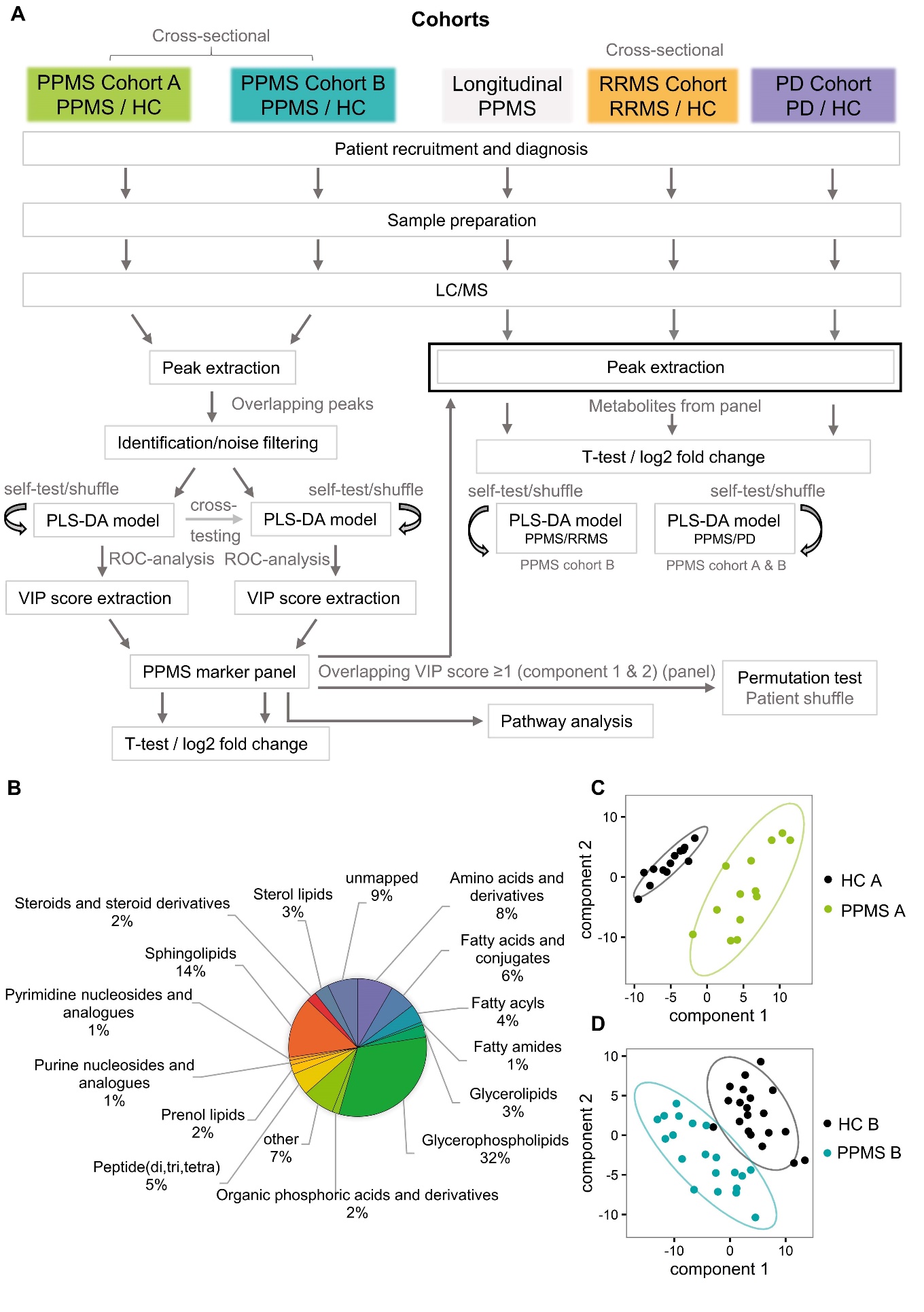
 Supplemental Figure 1: Workflow and summary of metabolomic profiling**. **(A)** General workflow in data analysis. **(B)** Pie chart illustrating the percentage of putatively identified metabolites from each metabolite class, classified according to KEGG, Lipidmaps and HMDB. In total 534 metabolites were analysed. **(C)** PLS-DA scores plot of all 534 metabolites in HC cohort A (n = 13) (black) and PPMS cohort A (n = 13) (green). **(D)** PLS-DA scores plot 534 metabolites identified in the HC cohort B (n = 20) (black) and PPMS cohort B (n = 20) (blue). Data in (C) and (D) show clear a separation between PPMS patients and HC. Ellipses assume a multivariate Gaussian distribution (2 sigma).

**
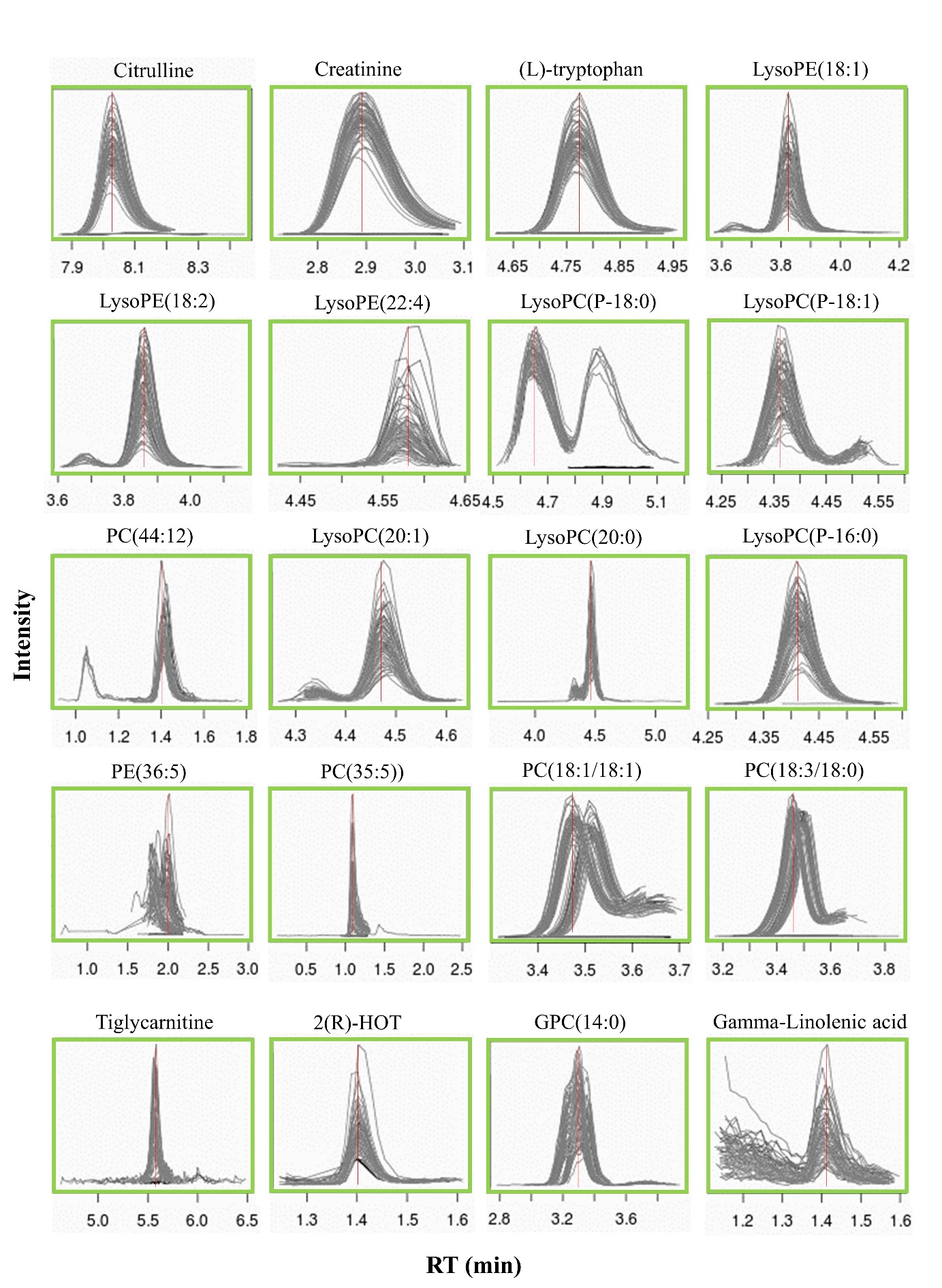
**

**Supplemental Figure 2: Extracted ion chromatograms of metabolites contributing to the specific PPMS signature.**

**
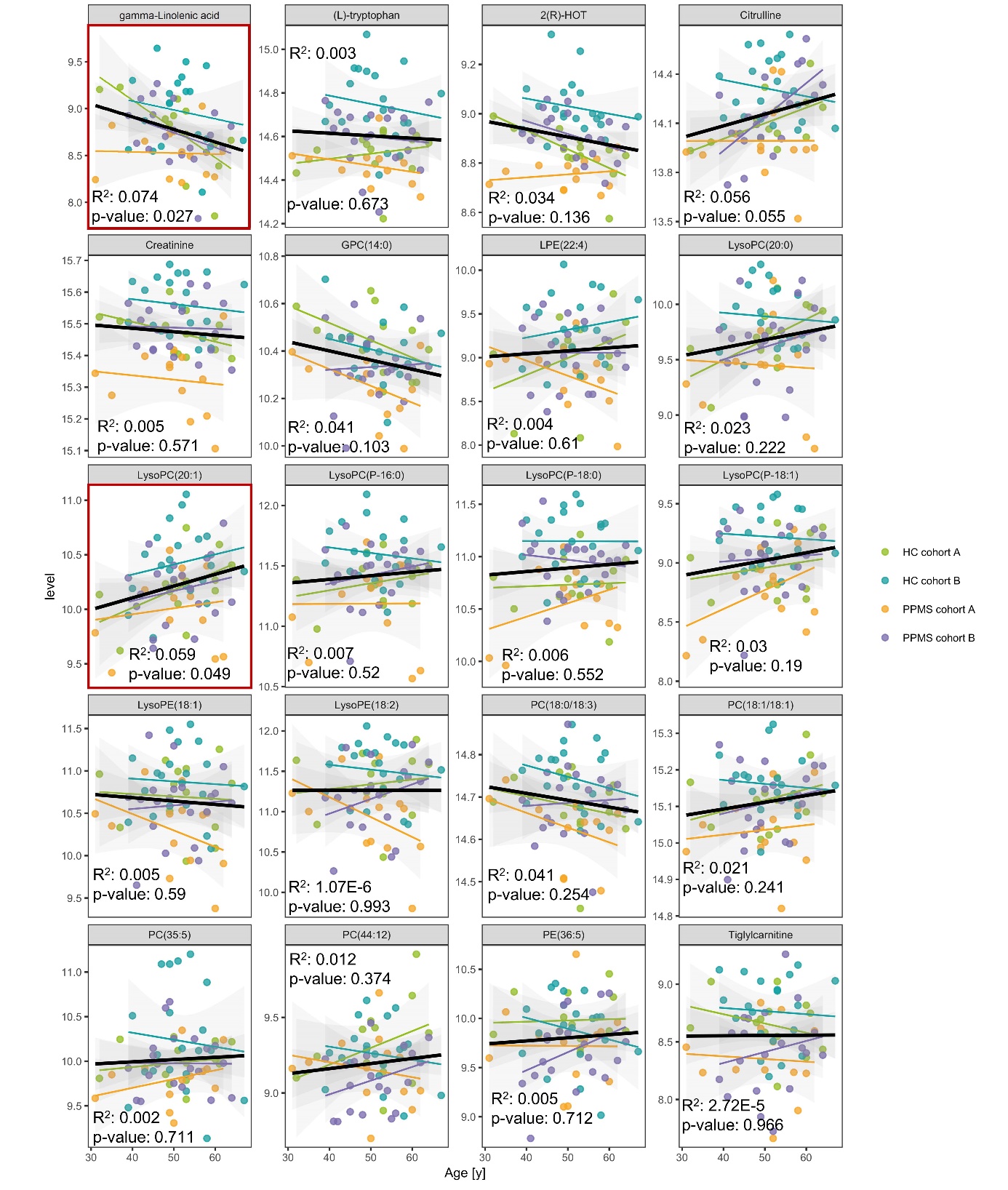
**

**Supplemental Figure 3: Linear model fit of PPMS marker levels and age of all analyzed individuals in PPMS cohorts A and B.** Utilized linear model to fit correlation between PPMS marker levels and ageing. Black: Linear model for all data points, light red: linear model for HC cohort A, green: linear model for HC cohort B, blue: linear model for PPMS cohort A, purple: linear model for PPMS cohort B. Grey areas around the lines indicate the 0.95 confidence interval. Metabolites with significant (p-value < 0.05) age and level correlations are highlighted with a red rectangle.

**
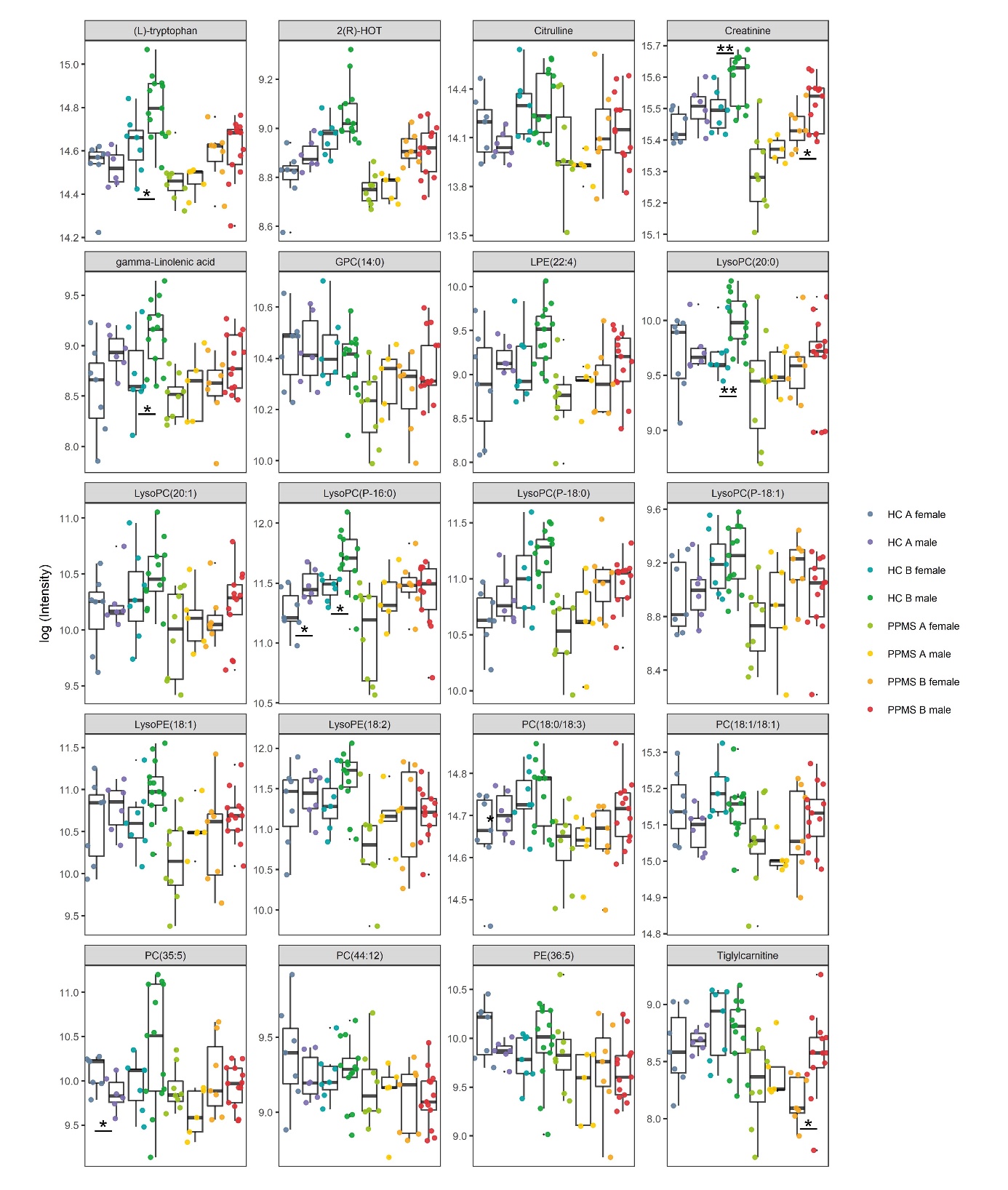
**

**Supplemental Figure 4: PPMS marker levels and sex dependency in all individuals analyzed.** Utilized one way ANOVA with Tukey’s post hoc test to compute differences between PPMS marker levels and sex in all groups. *Metabolites with significantly different levels between males and females (p-value < 0.05), ** Metabolites with significantly different levels between males and females (p-value < 0.01).


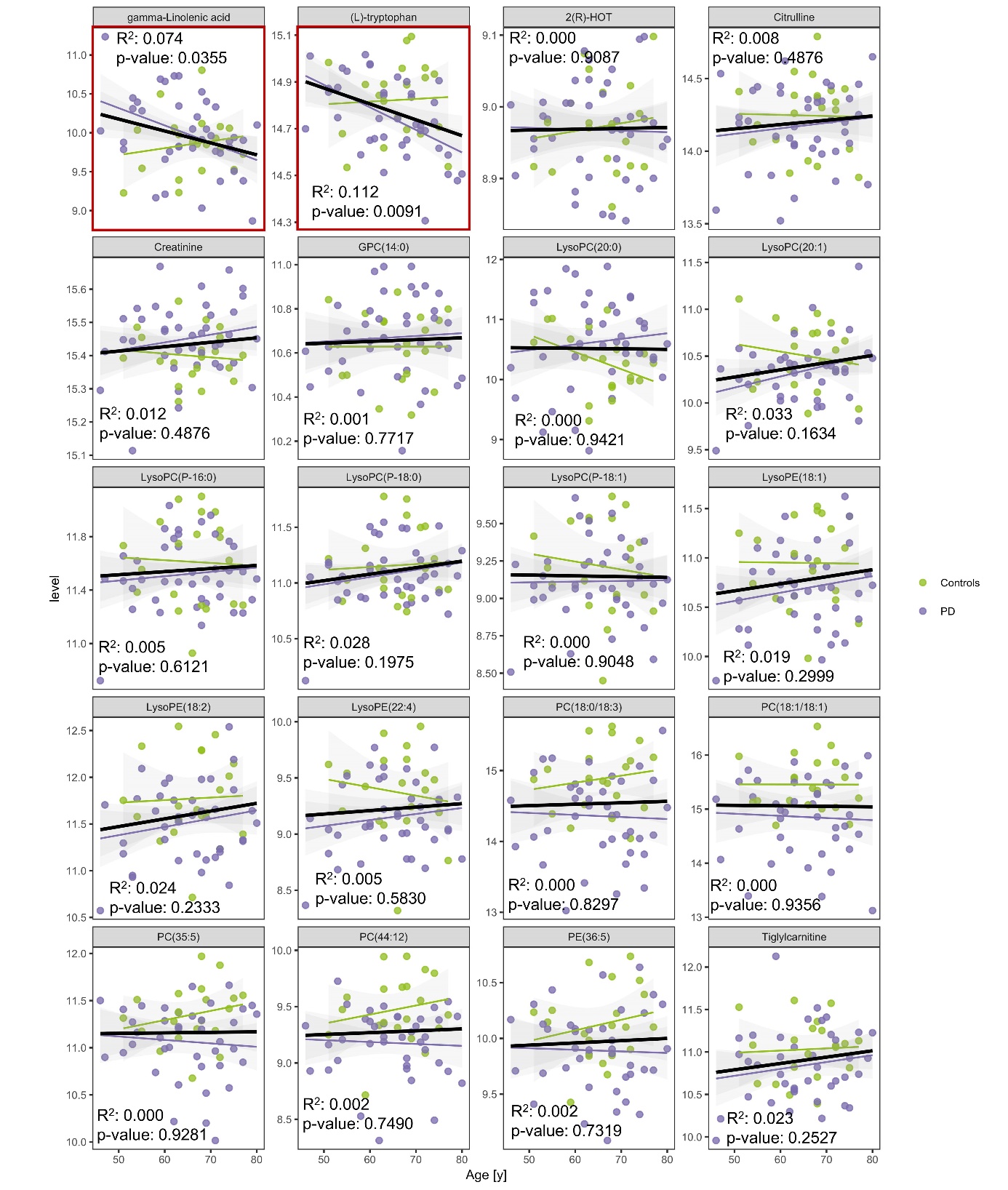


**Supplemental Figure 5: Linear model fit of PPMS marker levels and age of all individuals analyzed in the PD cohort.** Utilized linear model to fit correlation between PPMS marker levels and ageing. Black: Linear model for all data points, green: linear model for HC, purple: linear model for PD. Grey areas around the lines indicate the 0.95 confidence interval. Metabolites with significant (p-value < 0.05) correlations between age and level are highlighted with a red rectangle.

**
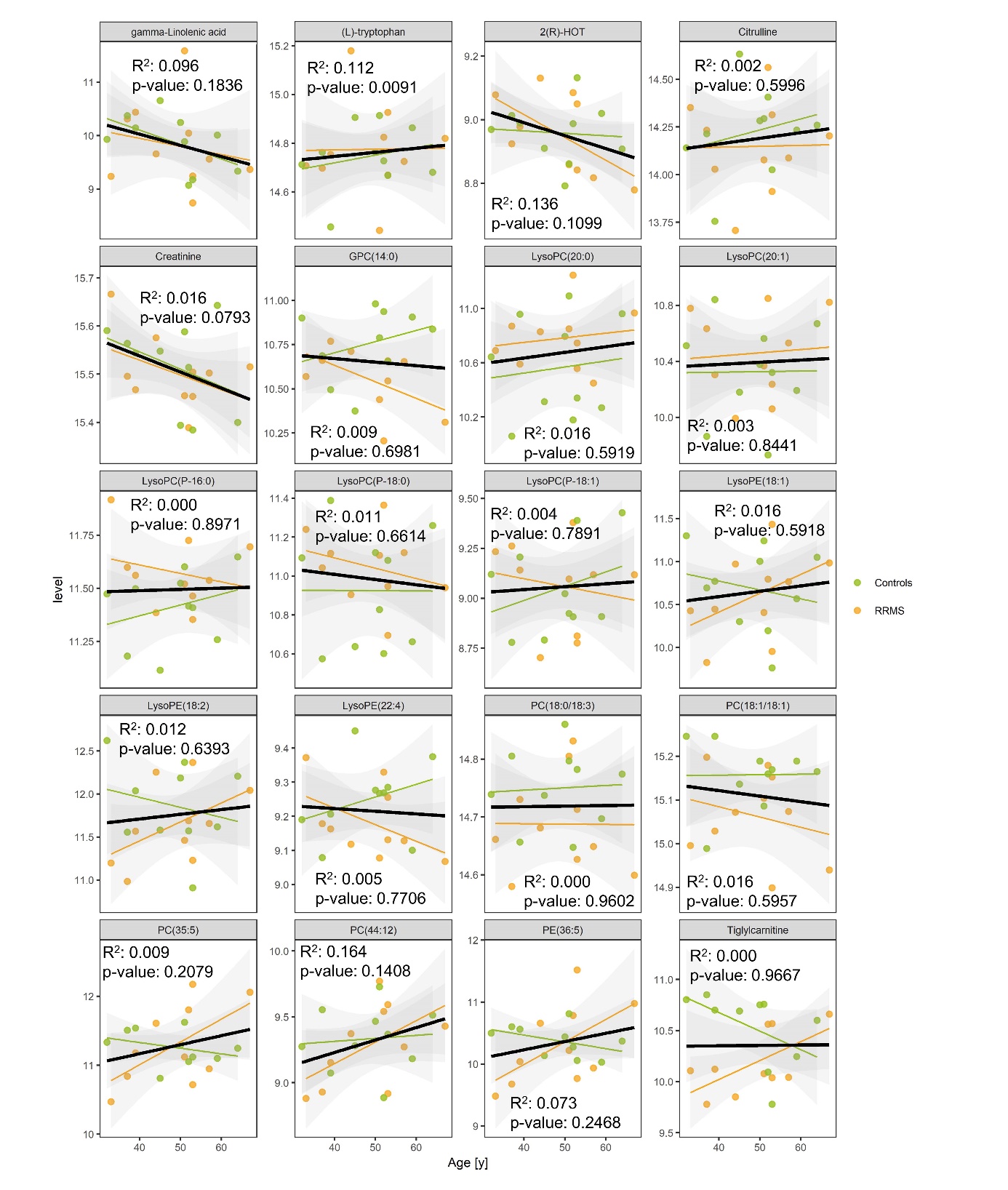
**

**Supplemental Figure 6: Linear model fit of PPMS marker levels and age of all analyzed individuals in the RRMS cohort.** Utilized linear model to fit correlation between PPMS marker levels and ageing. Black: Linear model for all data points, green: linear model for HC, orange: linear model for RRMS. Grey areas around the lines indicate the 0.95 confidence interval. Metabolites with significant (p-value < 0.05) age and level correlations are highlighted in a red rectangle.

**
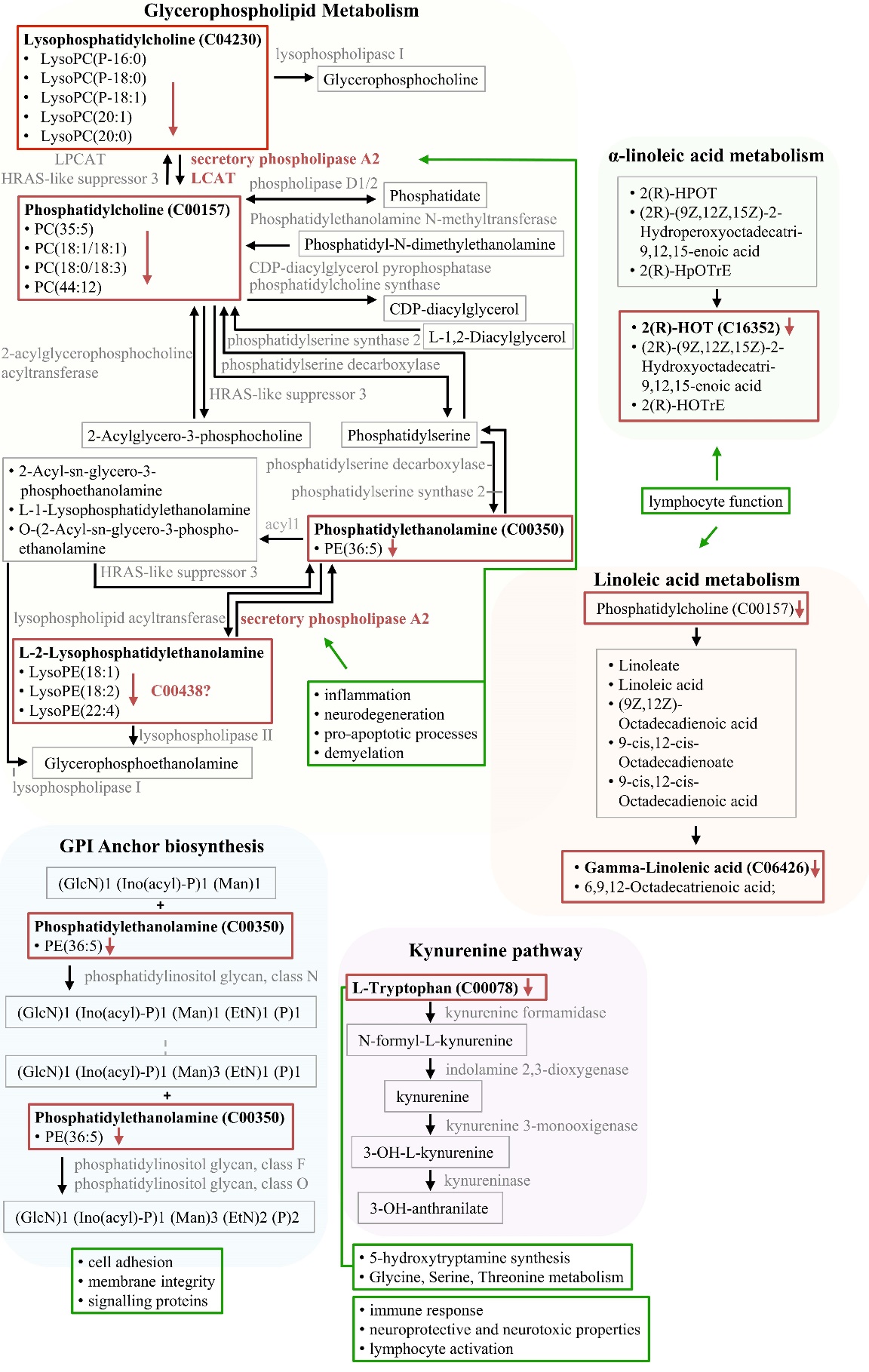
**

**Supplemental Figure 7: Pathway analysis of identified changes in PPMS patients compared to healthy controls (HC).** Pathway information retrieved from KEGG. Significantly altered metabolites are highlighted in dark red rectangles including the corresponding KEGG ID. Grey font indicates involved enzymes, dashed grey line indicates additional reactions which are not displayed. The substrates and products of reactions are shown in grey rectangular boxes. Green boxes indicate possible cellular function of observed changes in each pathway, decreased levels of metabolites compared to HC are indicated by blue arrows. Possible products and substrates are only partially displayed. Lysophasphatidylcholines lysoPE(18:1), lysoPE(18:2) and lysoPE(22:4) without reported KEGG ID (proposed KEGG ID C00438).

**Supplemental Table 2**: Inter-cohort dependencies determined by chi-square test for gender and one-way ANOVA for age, EDSS and disease duration (DD). Comparisons with signifcant p-values (p-value < 0.05) are highlighted in bold red.

| Comparison | sex  p-value | age  p-value | DD  p-value | EDSS  p-value |
| --- | --- | --- | --- | --- |
| PPMS cohort A vs. PPMS cohort B | 0.1185 | 0.9999 | / | / |
| PPMS patients cohort A vs. HC cohort A | 1 | 0.9999 | / | / |
| PPMS patients cohort B vs. HC cohort B | 1 | 1 | / | / |
| PPMS patients cohort A vs. PPMS patients cohort B | 0.255 | 0.7847 | 0.5459 | 0.2633 |
| HC cohort A vs. HC cohort B | 0.4778 | 0.8972 | / | / |
| RRMS patients vs. HC RRMS cohort | 0.0736 | 1 | / | / |
| PD patients vs. HC PD cohort | 0.9252 | 0.4987 | / | / |
| PPMS cohort A vs. RRMS cohort | 0.5829 | 0.8217 | / | / |
| PPMS cohort A vs. PD cohort | 0.1545 | **4.60 x 10^-9^** | / | / |
| PPMS cohort B vs. RRMS cohort | **0.0221** | 0.7188 | / | / |
| PPMS cohort B vs. PD cohort | 0.8991 | **2.39 x 10^-7^** | / | / |
| PPMS patients cohort A vs. PD patients | 0.3008 | **0.0059** | 0.3196 | / |
| PPMS patients cohort B vs. PD patients | 0.9252 | **0.0019** | 0.4923 | / |
| PPMS patients cohort A vs. RRMS patients | 1 | 0.9999 | 0.1634 | **0.0175** |
| PPMS patients cohort B vs. RRMS patients | 0.1547 | 0.9997 | 0.0838 | **0.0171** |
| Progression baseline vs. RRMS patients | 0.1648 | 0.6831 | **0.0231** | **0.0268** |
| Progression baseline vs. PD patients | 0.8874 | 0.4645 | 0.6177 | / |
| Progression baseline vs. RRMS patients cohort A | 0.2659 | 0.9209 | 0.6142 | 0.1850 |
| Progression baseline vs. RRMS patients cohort B | 1 | 0.9520 | 0.2789 | 0.7966 |

**Supplemental Table 3**: MS/MS fragments used for PPMS marker identification. Identities confirmed by available authentic standards (validation level 1). MS/MS spectra were matched against online databases such as Metlin and MassBank (validation level 2) or against *in silico* fragmentation spectra (validation level 3) retrieved from Metfrag, CFM-ID and/or CSI:FingerID with precursor mass accuracy of 20 ppm and fragment accuracy of 0.01 Da.

|  | **Proposed** |  | **positive ionization** | | **negative ionization** | | **Validation** |
| --- | --- | --- | --- | --- | --- | --- | --- |
| **Proposed metabolite** | **formula** | **m/*z*** | **Fragment *m/z*** | **Collision energy [V]** | **Fragment *m/z*** | **Collision energy [V]** | **level** |
| Citrulline | C_6_H_13_N_3_O_3_ | 175.09587 | 159.0763, 116.0711, 113.0712, 70.0661 | 10 | / | / | 1 |
| Creatinine | C_4_H_7_N_3_O | 113.05889 | 86.0752, 72.0479, 58.066, 113.9913 | 20 | / | / | 1 |
| (L)-tryptophan | C_11_H_12_N_2_O_2_ | 204.09020 | 147.0632, 119.069, 146.060, 166.095, 188.0711 | 20 | / | / | 1 |
| LysoPE(18:1) | C_23_H_46_NO_7_P | 479.30081 | 121.051, 283.175, 60.082 | 20 | 140.0127, 196.039, 281.249 | 20 | 3 |
| LysoPE(18:2) | C_23_H_44_NO_7_P | 477.28562 | 60.0815, 261.2214, 196.0342, 461.2653 | 10 | 184.0735, 280.237, 140.013, 279.233 | 20 | 3 |
| LysoPE(22:4) | C_27_H_48_NO_7_P | 529.31598 | 184.0733, 283.190, 205.195 | 20 | 59.015, 480.309 | 10 | 3 |
| / | / | / | / | / | 152.9968, 478.293, 256.238 | 20 | / |
| LysoPC(P-16:0) | C_24_H_50_NO_6_P | 479.33765 | 184.0735, 88.115, 223.2425 | 10 | 183.013, 283.265, 311.169, 312.171 | 10 | 3 |
| LysoPC(P-18:0) | C_26_H_54_NO_6_P | 507.36820 | 184.0735, 88.1132, 325.3104 | 10 | 265.1487, 59.015, 267.2695 | 10 | 3 |
| LysoPC(P-18:1) | C_26_H_52_NO_6_P | 505.35284 | 184.0735, 88.1131, 323.2955 | 10 | 265.1487, 266.152, 339.199, 183.013 | 10 | 3 |
| PC(44:12) | C_52_H_80_NO_8_P | 877.56643 | 184.074 | 10 | 59.015, 168.044, 480.309, 311.169 | 20 | 3 |
| LysoPC(20:1) | C_28_H_56_NO_7_P | 549.37870 | 184.0735, 88.1123, 293.2841, 550.3868 | 10 | 309.2801, 224.070, 291.2691 | 10 | 3 |
| LysoPC(20:0) | C_28_H_58_NO_7_P | 551.39427 | 295.299,184.0735, 104.1073, 88.1123 | 10 | 311.2957,293.2851, 267.3058 | 10 | 3 |
| PE(36:5) | C_41_H_72_NO_8_P | 737.49806 | 124.087, 601.518 | 20 | 140.013 | 10 | 3 |
| PC(35:5) | C_43_H_76_NO_8_P | 765.53003 | 184.074 | 20 | 87.0092, 79.958 | 10 | 3 |
| PC(18:1/18:1) | C_44_H_84_NO_8_P | 785.59391 | 184.0735, 504.6619, 728.0131 | 10 | 281.2491, 263.2385 | 10 | 3 |
| PC(18:0/18:3) | C_44_H_82_NO_8_P | 783.57697 | 184.0735, 267.2671, 88.1119 | 20 | 265.1487, 283.2647 | 20 | 3 |
| Tiglylcarnitine | C_12_H_21_NO_4_ | 243.14724 | 85.06479, 58.005, 60.081, 146.118, 188.092 | 20 | / | / | 3 |
| 2(R)-HOT | C_18_H_30_O_3_ | 294.22165 | 85.0284, 103.039, 146.094, 184.109 | 30 | / | / | 3 |
| GPC(14:0) | C_46_H_78_NO_7_P | 787.54959 | 86.09643, 184.073, 729.485, 605.493 | 10 | / | / | 3 |
| Gamma-Linolenic acid | C_18_H_30_O_2_ | 278.22449 | / | / | 209.1145, 178.0281 | 10 | 2 |
| **/** | / | / | / | / | 248.505 | 20 | / |

**Supplemental Table 5**: Model parameters for the tested models and their corresponding p-values after sample-label permutation test.

| Model | Q2 | R^2^Y | R^2^X | AUC | NPV | PPV | Accuracy |
| --- | --- | --- | --- | --- | --- | --- | --- |
| PPMS cohort A self-test | 0.68 | 0.98 | 0.24 | 79% (75%-82%) | 0.84 (0.80-0.88) | 0.75 (0.72-0.78) | 0.76 (0.74-0.79) |
| PPMS cohort A self-test shuffle  p-value | / | / | / | 55% (54%-56%)  0 | 0.50 (0.49-0.52)  0 | 0.50 (0.49-0.52)  0 | 0.50 (0.49-0.52)  0 |
| PPMS cohort B self-test | 0.54 | 0.86 | 0.09 | 73% (72%-74%) | 0.70 (0.66-0.73) | 0.69 (0.66-0.72) | 0.68 (0.66-0.71) |
| PPMS cohort B self-test shuffle  p-value | / | / | / | 54% (53%-54%)  0 | 0.50 (0.49-0.51)  0 | 0.50 (0.49-0.51)  0 | 0.50 (0.49-0.51)  0 |
| PPMS cohort B tested on PPMS cohort A | / | / | / | 70% (48%-80%) | 0.65 | 0.65 | 0.65 |
| PPMS cohort B tested on PPMS cohort A shuffle  p-value | / | / | / | 52% (51%-52%)  0.01 | 0.50 (0.49-0.51)  0.01 | 0.50 (0.50-0.51)  0 | 0.50 (0.50-0.51)  0 |
| PPMS vs RRMS | 0.93 | 0.93 | 0.79 | 78% (75%-82%) | 0.77 (0.73-0.81) | 0.72 (0.68-0.76) | 0.68 (0.65-0.71) |
| PPMS vs RRMS shuffle  p-value | / | / | / | 52% (52%-54%)  0 | 0.44 (0.43-0.46)  0.01 | 0.57 (0.56-0.58)  0.01 | 0.50 (0.49-0.52)  0 |
| PPMS vs PD | 0.56 | 0.56 | 0.69 | 80% (78%-82%) | 0.71 (0.68-0.73) | 0.75 (0.73-0.77) | 0.71 (0.70-0.73) |
| PPMS vs PD shuffle  p-value | / | / | / | 52% (51%-52%)  ≤ 0.001 | 0.45 (0.44-0.46)  ≤ 0.001 | 0.54 (0.53-0.55)  ≤ 0.001 | - 1. 0.50-0.51)   ≤ 0.001 |

**Supplemental Table 6**: Metabolites with significant changes between HC and PPMS patients (A and B cohorts) their corresponding AUC values and VIP score ranks for component 1 and 2. Univariate AUCs and 95% CI (500 bootstrappings) calculated using MetaboAnalyst.

| **Proposed metabolite** | **Proposed** | **AUC** | **AUC** | **95% CI** | **95% CI** | **VIP comp 1 A** | **VIP comp 1 B** | **VIP comp 2 A** | **VIP comp 2 B** |
| --- | --- | --- | --- | --- | --- | --- | --- | --- | --- |
|  | **formula** | **A** | **B** | **A** | **B** | **rank** | **rank** | **rank** | **rank** |
| Citrulline | C_6_H_13_N_3_O_3_ | 0.74 | 0.69 | 0.53-0.92 | 0.51-0.83 | 129 | 62 | 145 | 83 |
| Creatinine | C_4_H_7_N_3_O | 0.89 | 0.72 | 0.72-1.00 | 0.56-0.87 | 6 | 22 | 8 | 50 |
| (L)-tryptophan | C_11_H_12_N_2_O_2_ | 0.73 | 0.74 | 0.51-0.92 | 0.57-0.87 | 139 | 13 | 165 | 28 |
| LysoPE(18:1) | C_23_H_46_NO_7_P | 0.73 | 0.69 | 0.5-0.92 | 0.49-0.84 | 38 | 80 | 37 | 127 |
| LysoPE(18:2) | C_23_H_44_NO_7_P | 0.73 | 0.73 | 0.53-0.91 | 0.54-0.88 | 39 | 23 | 35 | 45 |
| LysoPE(22:4) | C_27_H_48_NO_7_P | 0.73 | 0.68 | 0.51-0.90 | 0.53-0.83 | 145 | 60 | 170 | 62 |
| LysoPC(P-16:0) | C_24_H_50_NO_6_P | 0.62 | 0.67 | 0.41-0.82 | 0.49-0.82 | 74 | 132 | 45 | 133 |
| LysoPC(P-18:0) | C_26_H_54_NO_6_P | 0.66 | 0.68 | 0.42-0.86 | 0.51-0.84 | 51 | 103 | 53 | 148 |
| LysoPC(P-18:1) | C_26_H_52_NO_6_P | 0.66 | 0.68 | 0.41-0.87 | 0.52-0.84 | 32 | 117 | 43 | 108 |
| PC(44:12) | C_52_H_80_NO_8_P | 0.66 | 0.72 | 0.44-0.87 | 0.56-0.88 | 115 | 25 | 133 | 54 |
| LysoPC(20:1) | C_28_H_56_NO_7_P | 0.65 | 0.71 | 0.43-0.85 | 0.53-0.85 | 36 | 84 | 30 | 156 |
| LysoPC(20:0) | C_28_H_58_NO_7_P | 0.7 | 0.69 | 0.48-0.91 | 0.51-0.84 | 53 | 122 | 70 | 81 |
| PE(36:5) | C_41_H_72_NO_8_P | 0.74 | 0.68 | 0.51-0.92 | 0.52-0.86 | 71 | 108 | 75 | 203 |
| PC(35:5) | C_43_H_76_NO_8_P | 0.69 | 0.61 | 0.48-0.89 | 0.42-0.78 | 132 | 88 | 121 | 87 |
| PC(18:1/18:1) | C_44_H_84_NO_8_P | 0.75 | 0.63 | 0.54-0.91 | 0.45-0.79 | 116 | 29 | 104 | 29 |
| PC(18:0/18:3) | C_44_H_82_NO_8_P | 0.7 | 0.71 | 0.46-0.88 | 0.54-0.86 | 47 | 131 | 40 | 115 |
| Tiglylcarnitine | C_12_H_21_NO_4_ | 0.75 | 0.76 | 0.54-0.92 | 0.61-0.90 | 28 | 9 | 22 | 15 |
| 2(R)-HOT | C_18_H_30_O_3_ | 0.84 | 0.79 | 0.64-1.00 | 0.53-0.84 | 10 | 17 | 9 | 11 |
| GPC(14:0) | C_46_H_78_NO_7_P | 0.81 | 0.66 | 0.62-0.96 | 0.46-0.81 | 143 | 7 | 136 | 6 |
| Gamma-Linolenic acid | C_18_H_30_O_2_ | 0.7 | 0.7 | 0.46-0.89 | 0.53-0.84 | 68 | 120 | 51 | 140 |

**Supplemental Table 7**: PPMS marker in PPMS patients compared to RRMS and PD patients. First p-value is determined by one-way ANOVA with Tukey’s post hoc with correction for multiple testing per metabolite. Metabolites with p-values < 0.05 are highlighted in bold green. Second p-value has been corrected (FDR) for all comparison including all metabolites.

|  | **p-value** | **log2 FC** | **p-value** | **log2 FC** | **p-value** | **log2 FC** | **p-value** | **log2 FC** | **p-value** | **log2 FC** |
| --- | --- | --- | --- | --- | --- | --- | --- | --- | --- | --- |
| **Metabolite** | **A vs B** | **A / B** | **A vs. RRMS** | **A / RRMS** | **B vs. RRMS** | **B / RRMS** | **A vs. PD** | **A / PD** | **B vs. PD** | **B / PD** |
| (L)-tryptophan | 0.8674 | 0.054 | 0.1679  0.5956 | -0.213 | **0.0202**  0.1413 | -0.267 | 0.1477  0.6714 | -0.165 | **0.0041**  0.0827 | -0.220 |
| 2(R)-HOT | 0.9996 | 0.001 | 0.6812  1 | -0.063 | 0.5611  1 | -0.064 | 0.2014  0.6714 | -0.075 | 0.0756  0.3025 | -0.077 |
| Citrulline | 0.9565 | 0.070 | 0.9522  1 | 0.085 | 0.9996  1 | 0.015 | 0.9829  0.9896 | 0.033 | 0.9954  0.9954 | -0.037 |
| Creatinine | 0.4011 | -0.078 | 0.3379  0.8448 | -0.099 | 0.9803  1 | -0.021 | 0.9282  0.9896 | -0.029 | 0.5463  0.9954 | 0.049 |
| Gamma-Linolenic acid | 0.9667 | -0.144 | 0.9906  1 | -0.547 | 0.9996  1 | -0.403 | 0.4700  0.8410 | -0.448 | 0.6956  0.9954 | -0.304 |
| GPC(14:0) | 0.5137 | -0.122 | 0.9973  1 | 0.014 | 0.4506 | 0.136 | 0.2372  0.6776 | -0.158 | 0.9760  0.9954 | -0.036 |
| LysoPC(20:0) | 0.9951 | -0.045 | **0.0367**  0.2754 | -0.895 | **0.0353**  0.1413 | -0.850 | **0.0308**  0.3346 | -0.988 | **0.0204**  0.1576 | -0.943 |
| LysoPC(20:1) | 0.9905 | -0.030 | 0.3168  0.8448 | -0.328 | 0.3848  1 | -0.298 | 0.6495  0.8410 | -0.185 | 0.7670  0.9954 | -0.155 |
| LysoPC(P-16:0) | 0.8019 | -0.082 | 0.1787  0.5956 | -0.272 | 0.5071  1 | -0.190 | 0.1821  0.6714 | -0.218 | 0.6283  0.9954 | -0.136 |
| LysoPC(P-18:0) | 0.9928 | -0.008 | 0.7824  1 | -0.103 | 0.8669  1 | -0.095 | 0.3518  0.8410 | -0.182 | 0.3961  0.9954 | -0.174 |
| LysoPC(P-18:1) | 0.9892 | -0.019 | 0.9643  1 | -0.037 | 0.9958  1 | -0.019 | 0.6274  0.8410 | -0.124 | 0.7542  0.9954 | -0.106 |
| LysoPE(18:1) | 0.9625 | -0.095 | 0.9724  1 | -0.132 | 1  1 | -0.037 | 0.6375  0.8410 | -0.225 | 0.8749  0.9954 | -0.129 |
| LysoPE(18:2) | 0.9867 | -0.038 | **0.0290**  0.2754 | -0.733 | **0.0345**  0.1413 | -0.695 | **0.0335**  0.3346 | -0.519 | **0.0315**  0.1576 | -0.481 |
| LysoPE(22:4) | 0.9951 | -0.055 | 0.6821  1 | -0.148 | 0.7564  1 | -0.093 | 0.6508  0.8410 | -0.167 | 0.7239  0.9954 | -0.112 |
| PC(18:0/18:3) | 1.0000 | -0.001 | 1.0000  1 | -0.002 | 1  1 | -0.001 | 0.0751  0.5004 | 0.249 | **0.0266**  0.1576 | 0.250 |
| PC(18:1/18:1) | 1.0000 | -0.008 | 0.9976  1 | 0.060 | 0.9957  1 | 0.067 | 0.4386  0.8410 | 0.046 | 0.2849  0.9498 | 0.053 |
| PC(35:5) | 0.9977 | -0.058 | 0.7927  1 | -0.378 | 0.8390  1 | -0.320 | 0.9469  0.9896 | 0.079 | 0.8158  0.9954 | 0.137 |
| PC(44:12) | 0.9030 | 0.108 | 0.9997  1 | -0.037 | 0.8860  1 | -0.145 | 0.6728  0.8410 | 0.124 | 0.9737  0.9954 | 0.016 |
| PE(36:5) | 1.0000 | 0.028 | 0.0551  0.2754 | -0.829 | **0.0327**  0.1413 | -0.857 | 0.9896  0.9896 | -0.027 | 0.9867  0.9954 | -0.055 |
| Tiglylcarnitine | 0.9645 | -0.144 | **0.0489**  0.2754 | 0.730 | **0.0079**  0.1413 | 0.874 | 0.5350  0.8410 | -0.295 | 0.7751  0.9954 | -0.151 |
